# Hydrophobic unnatural base pair promotes trigger loop closure and catalysis in cellular RNA polymerase independent of hydrogen bonding

**DOI:** 10.1073/pnas.2607774123

**Published:** 2026-08-12

**Authors:** Qingrong Li, Peini Hou, Michiko Kimoto, Juntaek Oh, Yan Liu, Hui Pen Tan, Jenny Chong, Ichiro Hirao, Dong Wang

**Affiliations:** ^a^ Department of Pharmaceutical Sciences, Skaggs School of Pharmacy and Pharmaceutical Sciences, University of California San Diego, La Jolla, CA 92093; ^b^Xenolis Pte. Ltd., Singapore 118259, Singapore; ^c^ Department of Regulatory Science, Graduate School, Kyung Hee University, Seoul 02447, Republic of Korea; ^d^ Institute of Regulatory Innovation through Science, Kyung Hee University, Seoul 02447, Republic of Korea; ^e^ Institute of Integrated Pharmaceutical Sciences, Kyung Hee University, Seoul 02447, Republic of Korea; ^f^ Division of Cryo-electron Microscopy and Bioimaging, Stanford Synchrotron Radiation Lightsource, SLAC National Accelerator Laboratory, Stanford University, Menlo Park, CA 94025-7015; ^g^ Department of Molecular and Cellular Medicine, School of Medicine, University of California San Diego, La Jolla, CA 92093; ^h^ Department of Biochemistry and Molecular Biophysics, University of California San Diego, La Jolla, CA 92093

**Keywords:** transcription, unnatural base pair, trigger loop, hydrogen bond, synthetic biology

## Abstract

Expanded genetic alphabets based on synthetic nucleotides offer a powerful strategy to increase the chemical diversity and functional potential of nucleic acids. Hydrophobic unnatural base pairs (UBPs) represent a unique class of UBPs that operate without canonical hydrogen bonding and shapes, which are distinct from natural base pairs. However, the molecular mechanisms governing hydrophobic UBP recognition and incorporation by RNA polymerases remain poorly understood, because no structures of catalytically competent state have been captured. Here, we investigate the enzyme kinetics and structural basis of transcription recognition of hydrophobic 7-(2-thienyl)-imidazo[4,5-b]pyridine (Ds):pyrrole 2-carbaldehyde (Pa) base pair by the *Escherichia coli* RNA polymerase (*Ec*RNAP). Enzyme kinetics reveal a pronounced strand-dependent asymmetry, with DsTP incorporated substantially more efficiently than PaTP. To elucidate the structural origin of this asymmetry, we determined two cryo-electron microscopy structures of *E. coli* RNAP elongation complexes containing dPa:DsTP and dDs:PaTP at resolutions of 3.20 Å and 3.24 Å, respectively. The dPa:DsTP structure captures a pre-catalytic state of hydrophobic UBP transcription in which DsTP forms an edge-to-edge base pair with templating dPa, and the active site adopts a native-like, catalytically competent configuration with fully folded trigger loop. In contrast, the dDs:PaTP structure reveals a pre-insertion intermediate with incomplete substrate loading. Structural modeling indicates that PaTP must undergo an additional insertion step to achieve productive catalysis. Together, these findings define the molecular basis of asymmetric Ds:Pa UBP transcription recognition by a multisubunit RNAP and provide structural principles for designing next-generation UBPs compatible with cellular transcription.

## Significance

The recognition of natural Watson–Crick base pairs between substate and its DNA template by RNA polymerases is based on the specific hydrogen bonding patterns and the geometric shape compatibility. Hydrophobic unnatural base pairs (UBPs) represent a unique class that operates with distinct chemical principles without canonical hydrogen-bond complementarity. However, the molecular mechanisms by which hydrophobic UBP substrates are recognized and incorporated by RNA polymerases remain poorly understood. Here we systematically investigate the enzymatic, kinetic, and structural basis of transcription recognition of the hydrophobic Ds:Pa base pair by the multisubunit *Escherichia coli* RNA polymerase. Our findings reveal that hydrogen bonding between base pairs is not required to trigger active-site conformational changes in cellular RNA polymerase during catalysis.

**Fig. 1.**
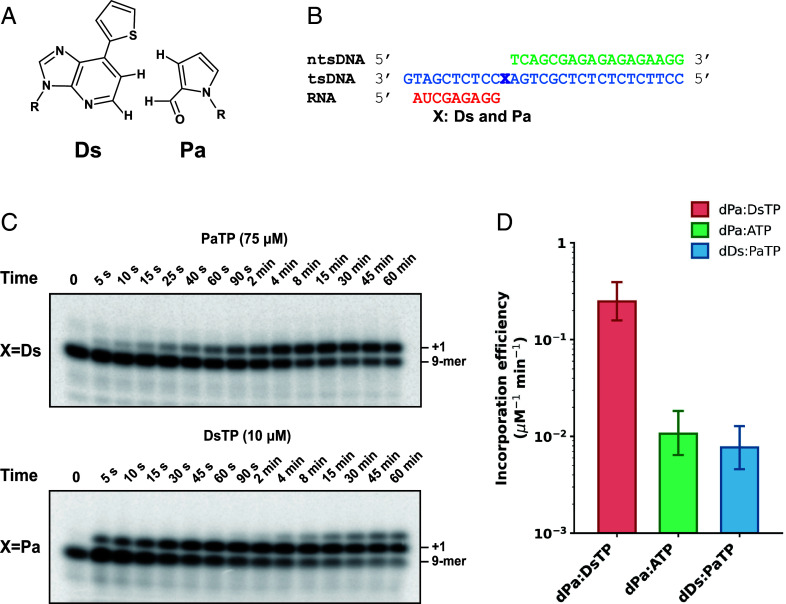
Single-nucleotide transcription and kinetic analyses reveal asymmetric incorporation of the Ds:Pa base pair by *E. coli* RNA polymerase. (*A*) Chemical structures of the Ds:Pa unnatural base pair used in this study. (*B*) Nucleic acid scaffold used for transcription assays, consisting of tsDNA (cyan), ntsDNA (green), and RNA (red). “X” denotes the templating position (*i*+1) containing either dDs or dPa. (*C*) Single-nucleotide transcription assays testing incorporation of PaTP (*Upper*) and DsTP (*Lower*) opposite cognate dDs or dPa in the template strand. Because PaTP exhibits substantially lower incorporation efficiency, a higher PaTP concentration (75 μM) was used compared with DsTP (10 μM). Reactions were sampled at 14 time points ranging from 5 s to 60 min. Transcription products were analyzed by denaturing PAGE and visualized by ^32^P-labeled 9-mer RNA (5′-AUGGAGAGG-3′). The 0-s band corresponds to the 9-mer RNA and serves as an internal size marker; bands labeled “+1” indicate extended RNA products. (*D*) Kinetic analyses of cognate incorporations (dPa:DsTP and dDs:PaTP) and misincorporation reaction (dPa:ATP), displayed by the defined incorporation efficiency constant k*_pol_max_*/K*_d_app_*. Error bars represent the 95% CI (profile likelihood) of the fitted parameters. Numerical values are summarized in Table 1.

Expanding the natural four-letter genetic code with orthogonal unnatural base pairs has been a central objective in synthetic biology, offering a route to diversify nucleic acid chemistry beyond natural constraints (1–3). Pioneering efforts from the Benner, Romesberg, and Hirao groups have established multiple UBP systems that can be replicated and transcribed in vitro (1–3). Among these, the Ds:Pa base pair—composed of 7-(2-thienyl)imidazo[4,5-b]pyridine (Ds) and pyrrole-2-carbaldehyde (Pa)—represents a unique design strategy based on size complementarity and hydrophobic interactions rather than canonical Watson–Crick hydrogen bonding (Fig. 1*A*) (4). Ds was engineered to mimic the size and shape of a purine base and to incorporate a bulky thienyl substituent that sterically disfavors mispairing with native nucleotides (1, 4–6). In contrast, Pa functions as a smaller partner analogous to a pyrimidine, employing a five-membered pyrrole ring to achieve tight size complementarity while avoiding steric clashes (1, 4, 5, 7, 8). In addition, the aldehyde group of Pa mimics the 2-keto group of native pyrimidines, providing an extended conjugated π system for better base stacking that facilitates polymerase recognition despite the absence of hydrogen bonding (4, 8, 9).

Ds:Pa and its analogs have been successfully applied in DNA replication and transcription systems (9–17). In particular, the Ds:Pa system has been widely employed with T7 RNA polymerase for site-specific incorporation of functional substrates into RNA, including fluorescent dyes, biotin, and chemical probes via click chemistry, enabling applications in RNA labeling and molecular sensing (1, 4). Structural studies have revealed how Ds:Px (a Pa analog) is accommodated by DNA polymerases (18), and our previous work combined kinetic and crystallographic analyses to elucidate the transcription mechanism of Ds:Pa by T7 RNA polymerase, a single-subunit bacteriophage RNAP (19). However, the transcriptional mechanism of Ds:Pa in multisubunit RNA polymerases remains uncharacterized. Because multisubunit RNAPs carry out cellular transcription and use substrate-selection pathways fundamentally distinct from T7 RNA polymerase, defining their UBP recognition and incorporation mechanism is essential for extending Ds:Pa to cellular contexts. Moreover, despite previous investigations on hydrophobic UBPs in transcription, no pre-catalytic, catalytically competent UBP-bound state has been structurally captured in RNAP, leaving the recognition and activation mechanism of hydrophobic UBP substrates poorly understood (19, 20).

Here, we investigate the molecular mechanism of Ds:Pa transcription by *Escherichia coli* RNA polymerase (*E. coli* RNAP) using a combined approach integrating enzyme kinetics and cryoelectron microscopy. By reconstituting *E. coli* RNAP elongation complex (EC) in vitro, we quantitatively characterized the incorporation efficiency and asymmetry of Ds:Pa transcription and assessed its orthogonality relative to native nucleotides. We further determined cryo-EM structures of *E. coli* RNAP elongation complexes containing either dPa:DsTP or dDs:PaTP, capturing distinct substrate-loading states. Our results reveal that hydrophobic UBPs can be structurally observed to induce trigger loop folding and a native-like catalytic architecture in a multisubunit RNA polymerase, while also uncovering a pre-insertion intermediate that underlies asymmetric incorporation. Together, these findings provide mechanistic insight into UBP transcription by cellular RNA polymerases and establish structural principles to guide the rational design of next-generation hydrophobic UBPs.

## Results

### Efficient and Asymmetric Incorporation of Ds:Pa during *E. coli* RNA Polymerase Transcription.

To examine whether the hydrophobic Ds:Pa base pair can be accommodated by *E. coli* RNAP during transcription, we performed single-nucleotide incorporation assays using synthetic scaffolds containing a site-specific unnatural base (dDs or dPa) positioned at the *i*+1 site of the template strand (Fig. 1*B*). The corresponding complementary ribonucleotide triphosphates, PaTP and DsTP, were supplied individually to test their incorporation opposite the templating base by *E. coli* RNAP.

In both scaffolds, addition of the cognate synthetic nucleotide resulted in a clear band shift from the 9-nt RNA primer to the *n*+1 extended product, demonstrating that the Ds:Pa base pair can be efficiently incorporated during transcription by *E. coli* RNAP (Fig. 1*C*). These results indicate that Ds:Pa is compatible with the transcriptional machinery despite lacking canonical Watson–Crick hydrogen bonding.

A closer examination revealed pronounced asymmetry in incorporation efficiency between the two pairing orientations. Incorporation of PaTP opposite templating dDs was markedly slower than incorporation of DsTP opposite templating dPa. Notably, even when a substantially higher concentration of PaTP (75 μM) was used compared with DsTP (10 μM), PaTP incorporation required a longer reaction time to achieve comparable extension (Fig. 1*C*). These observations suggest an intrinsic asymmetry in Ds:Pa recognition and incorporation by *E. coli* RNAP.

To quantitatively characterize this asymmetry, we performed kinetic analyses for both dPa:DsTP and dDs:PaTP incorporation reactions (Fig. 1*D* and Table 1). Two kinetic parameters were determined: the catalytic rate constant (k*_pol_max_*) and the apparent dissociation constant (K*_d_app_*). The ratio k*_pol_max_*/K*_d_app_* was calculated as a measure of overall substrate incorporation efficiency. Raw kinetic traces and detailed fitting procedures are provided in *SI Appendix*, Figs. S1 and S2 and Dataset S1.

**Table 1.** Kinetic parameters of dPa:DsTP, dPa:ATP, and dDs:PaTP incorporation

	k*_pol___max_* (min^−1^)	K*_d_app_* (μM)	k*_pol___max_*/K*_d_app_* (μM^−1^ min^−1^)	Relative efficiency
dPa:DsTP	18.3 (16.9 to 19.8)	74 (51 to 107)	2.5 × 10^−1^	1
dPa:ATP	9.7 (8.5 to 11.5)	910 (630 to 1,330)	1.1 × 10^−2^	4.4 × 10^−2^
dDs:PaTP	3.5 (3.1 to 4.0)	450 (310 to 680)	7.8 × 10^−3^	3.1 × 10^−2^

Note: Values in parentheses indicate the 95% CI (profile likelihood) obtained from nonlinear regression fitting. k*_pol_max_* is the polymerization rate constant; K*_d_app_* is the apparent dissociation constant; incorporation efficiency is defined as k*_pol_max_*/K*_d_app_*; relative efficiency is normalized to the reference substrate.

Consistent with the qualitative transcription assays, Ds:Pa exhibited strongly asymmetric incorporation efficiency (Fig. 1*D* and Table 1). Incorporation of DsTP opposite templating dPa was nearly 30-fold more efficient than incorporation of PaTP opposite templating dDs. Specifically, the dPa:DsTP reaction displayed a ~five-fold higher catalytic rate (k*_pol_max_*) and a ~six-fold stronger apparent binding affinity (K*_d_app_*) compared with dDs:PaTP. A similar asymmetric incorporation pattern for Ds:Pa has been reported for T7 RNA polymerase (19), suggesting that this asymmetry reflects an intrinsic property of Pa as a substrate rather than a structural feature specific to *E. coli* RNAP. The mechanistic implications of this asymmetric recognition are analyzed in *Discussion* section.

We also noted minor extension products beyond the +1 position at late time points in the dPa:DsTP assay (Fig. 1*C*), most likely reflecting slow DsTP misincorporation at the downstream natural template position following cognate incorporation at dPa. However, cognate incorporation proceeds with substantially higher efficiency than this secondary misincorporation under the same conditions. As an intrinsic limitation of single-nucleotide transcription assays, the polymerase is forced to misincorporate in the absence of any competing substrate at the downstream position. The misincorporation observed here is therefore likely an overestimate of the actual error rate when the full complement of natural NTPs is present. The cognate natural substrate at the downstream position would kinetically outcompete this rare and slow event in a complete transcription system.

### Orthogonality of the Ds:Pa Base Pair Relative to Native Base Pairs.

To provide a comprehensive evaluation of Ds:Pa specificity and orthogonality, we performed two sets of single-nucleotide incorporation assays. First, to test how orthogonal Ds and Pa serve as a DNA template nucleobase, we assembled *Ec*RNAP containing either a site-specific dDs or dPa at the *i*+1 position and systematically measured the nucleotide incorporation for all possible six substrate NTPs (DsTP, PaTP, ATP, GTP, CTP, and UTP) (*SI Appendix*, Fig. S3*B*). In a second set of experiments, to test how orthogonal DsTP and PaTP serve as a substrate, we measure DsTP and PaTP incorporation against *Ec*RNAP containing each of the six template nucleobases (dDs, dPa, dA, dG, dC, and dT) at the *i*+1 position (*SI Appendix*, Fig. S3*C*).

Transcription results from both sets of experiments revealed that Ds exhibited high fidelity both as a template and a substrate. When dDs was present as a template base (*SI Appendix*, Fig. S3*B*), only PaTP was efficiently incorporated, with all other substrates showing only trace incorporation. When DsTP was supplied as the incoming substrate (*SI Appendix*, Fig. S3*C*), efficient incorporation was observed exclusively opposite dPa, with only trace misincorporation at any natural template position and no detectable dDs:DsTP self-pairing. These results demonstrate that Ds maintains high orthogonality whether acting as a template or substrate.

In contrast, Pa was associated with two notable misincorporation events, the Pa:A mismatch and the Pa:Pa self-mismatch (*SI Appendix*, Fig. S3 *B* and *C*). Both mismatches were observed regardless of whether Pa occupied the template or substrate position.

The Pa:A mismatch is consistent with previous reports for both T7 RNA polymerase-mediated transcription and DNA polymerase-catalyzed replication (4, 19). However, given the efficient cognate incorporation of dPa:DsTP and dA:UTP, the Pa:A mismatch would be expected to occur only rarely when it competes with the cognate substrate incorporation. To get a comparison of nucleotide incorporation efficiency, we performed detailed pre–steady state kinetic analysis comparing dPa:DsTP and dPa:ATP incorporation, respectively. We found that incorporation of cognate DsTP opposite templating dPa is strongly favored (Fig. 1*D* and Table 1). Specifically, the overall incorporation efficiency (k*_pol_max_*/K*_d_app_*) of dPa:DsTP is approximately 23-fold higher than that of the misincorporated dPa:ATP pair, a difference primarily driven by substrate binding, as reflected by an approximately 12-fold lower K*_d_app_* for DsTP compared with ATP. To directly test whether this kinetic preference is sufficient to suppress the Pa:A mismatch under competitive conditions, we performed a competition assay in which ECs assembled on a dPa template were incubated with 50 μM ATP together with increasing concentrations of DsTP (2.5 to 20 μM) (*SI Appendix*, Fig. S3*E*). DsTP outcompetes ATP for incorporation opposite dPa across all the concentration range we tested (even at a 20-fold molar excess of ATP), consistent with the strong kinetic preference for dPa:DsTP over dPa:ATP. Taken together, these results indicate that although the Pa:A mismatch can occur in the absence of DsTP, it is efficiently suppressed by the cognate DsTP under competitive substrate conditions.

A second misincorporation event is the dPa:PaTP self-pairing. Similar to the Pa:A mismatch, Pa:Pa self-pairing proceeds with lower efficiency than the cognate dPa:DsTP incorporation (*SI Appendix*, Fig. S3 *B*, *Lower*), suggesting that cognate DsTP would similarly outcompete PaTP for the dPa template. We confirmed this with a direct competition assay in which ECs assembled on a dPa template were incubated with 50 μM PaTP together with increasing concentrations of DsTP (2.5 to 20 μM) (*SI Appendix*, Fig. S3*D*). We observed substantial cognate DsTP incorporation even at a 20-fold molar excess of PaTP (2.5 μM DsTP vs. 50 μM PaTP). PaTP misincorporation is greatly suppressed in the presence of DsTP.

Beyond these two major mismatches, additional trace misincorporation events were also observed across the single-nucleotide incorporation assays (*SI Appendix*, Fig. S3 *B* and *C*). As discussed in the above section, a limitation of single substrate incorporation assays is to force the polymerase to misincorporate in the absence of competing cognate substrate, and therefore likely overestimates the extent of misincorporation. Consistent with this interpretation, the competition assays (for the Pa:A and Pa:Pa mismatches, *SI Appendix*, Fig. S3 *D* and *E*) show that these notable misincorporation events are efficiently outcompeted by cognate DsTP, suggesting that the additional minor misincorporation events in *SI Appendix*, Fig. S3 *B* and *C* would be similarly suppressed under competitive substrate conditions.

Taken together, these results demonstrate that, even in the presence of competing native NTPs, *E. coli* RNAP preferentially incorporates the cognate Ds:Pa pair. Thus, the Ds:Pa base pair maintains functional orthogonality during transcription under physiologically relevant substrate conditions.

### Cryo-EM Structures of *E. coli* RNAP EC Incorporating dPa:DsTP.

**Fig. 2.**
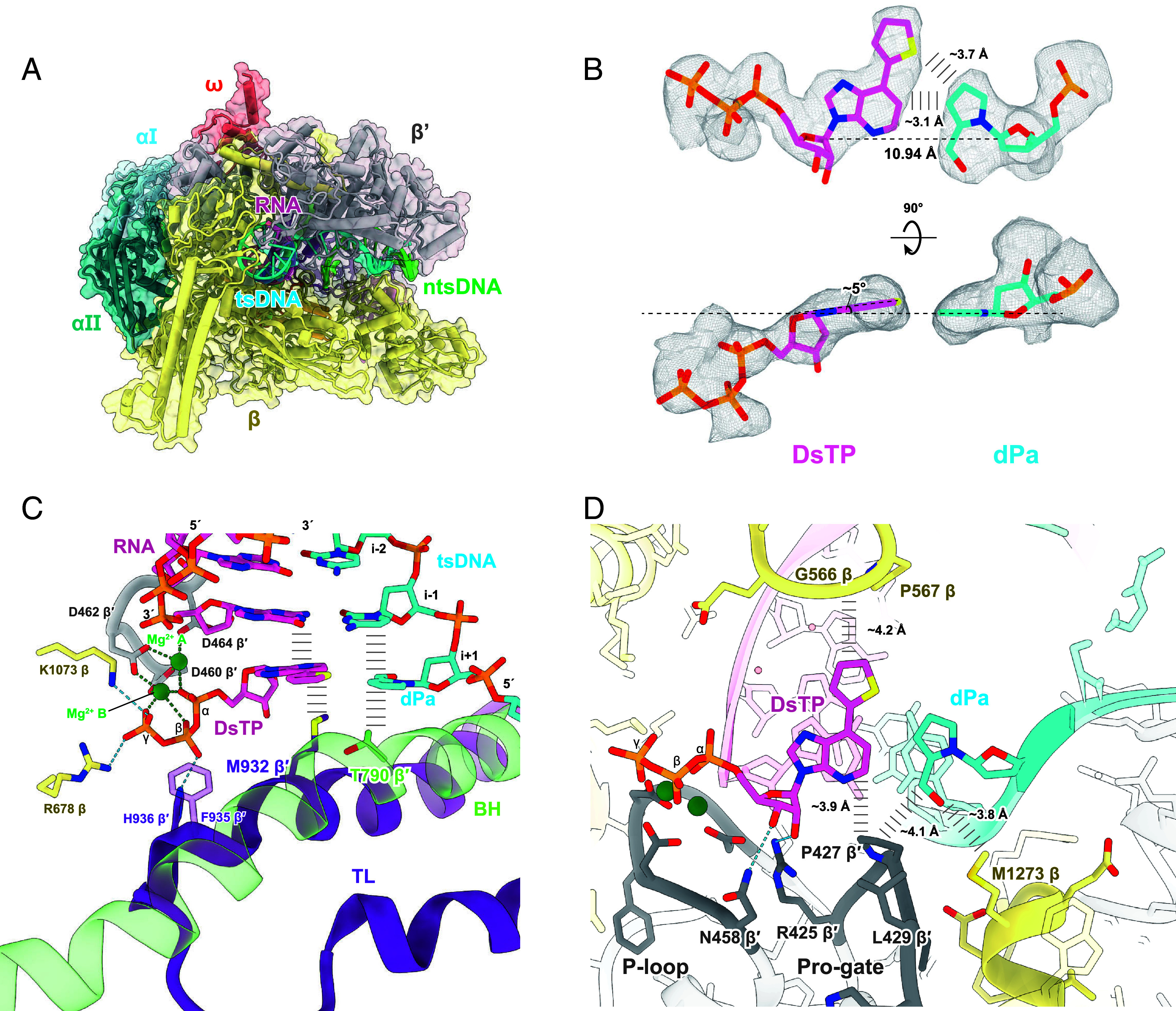
Cryo-EM structure of the *E. coli* RNA polymerase EC containing dPa:DsTP. (*A*) Overview of the cryo-EM structure of the *E. coli* RNA polymerase EC assembled with a templating dPa and the incoming DsTP substrate. The EC comprises five RNAP subunits—αI (light blue), αII (teal), β (yellow), β′ (silver), and ω (salmon)—together with the nucleic-acid scaffold composed of tsDNA (cyan), ntsDNA (green), and RNA (hot pink). (*B*) The incoming DsTP (hot pink) forms a size-complementary, edge-to-edge base pair with templating dPa (cyan). Cryo-EM density is shown as a silver mesh contoured at 4-σ. Hydrophobic contacts are indicated by gray bold, dashed lines with distances labeled. The C1′–C1′ distance between the paired bases is indicated, and the inter-base-plane angle (~5°) is shown in the *Lower* panel. (*C*) Architecture of the active site in the dPa:DsTP EC. Key structural elements include the RNA primer, tsDNA, the β′ P-loop (silver), TL (purple), two catalytic Mg^2+^ ions (green), and the BH (light green). Residues involved in substrate stabilization are shown as sticks. Hydrogen bonds are depicted as blue dashed lines, and hydrophobic interactions with the Ds:Pa nucleobases are shown as gray bold dashed lines. (*D*) Detailed interactions between RNAP and the Ds:Pa base pair at the major- and minor-groove edges. Hydrophobic interactions with the nucleobase moieties are indicated by gray bold dashed lines with distances labeled. Hydrogen bonds involving the 2′- and 3′-hydroxyl groups of the DsTP ribose are shown as blue dashed lines.

To understand how a multisubunit RNA polymerase recognizes and incorporates the hydrophobic Ds:Pa base pair, we determined a cryo-EM structure of an *E. coli* RNA polymerase EC containing a templating dPa paired with an incoming DsTP at the *i*+1 position (Fig. 2 and *SI Appendix*, Fig. S5 and Table S1). To trap a pre-catalytic state, the EC was reconstituted with a 3′-deoxy RNA primer, which permits substrate binding while preventing phosphodiester bond formation, thereby stabilizing a pre-catalysis intermediate. From this dataset, we obtained a cryo-EM reconstruction at a resolution of 3.20 Å, in which all five RNAP subunits (αI, αII, β, β′, and ω) are well resolved (Fig. 2*A* and *SI Appendix*, Fig. S5).

This structure captures a pre-catalytic EC in which the substrate DsTP is positioned immediately prior to nucleotide incorporation. The incoming DsTP forms an edge-to-edge base pair with the +1 dPa nucleobase at the DNA template strand (Fig. 2 *B* and *C*). Notably, the structure reveals folding of the trigger loop (TL; residues 916 to 1146 of the β′ subunit) into its α-helical conformation, a key domain transition that precedes phosphodiester bond formation during native transcription (Fig. 2*C*). Together, these features demonstrate that Ds:Pa pairing supports a native-like active-center architecture in a transcribing multisubunit RNAP, which has not previously been structurally characterized for hydrophobic unnatural base pairs. Detailed comparisons with other UBP-containing RNA polymerase complexes are presented in *Discussion* section (20).

### Incoming DsTP Forms an Edge-to-Edge Base Pair with Templating dPa.

The cryo-EM density corresponding to the Ds:Pa base pair is well resolved, allowing unambiguous modeling of the pairing geometry (Fig. 2*B*). The structure shows that Pa and Ds associate through the intended size-complementary mechanism, forming an edge-to-edge base pair with a C1′–C1′ distance of 10.9 Å and an inter-base-plane angle of approximately 5° (Fig. 2 *B* and *C*). The five-membered ring of dPa is positioned at distances of approximately 3.1 Å and 3.7 Å from the hydrophobic aromatic core and the thienyl group of Ds, respectively (Fig. 2*B*). The Ds:Pa base pair is oriented approximately parallel to the upstream nucleobase at the *i*–1 position (Fig. 2*C*). Within the active site, the Ds base stacks against TL residue M932 and the RNA nucleobase at *i*–1, whereas the Pa base stacks against bridge helix residue T790 and the templating DNA base at *i*–1 (Fig. 2*C*).

Additional hydrophobic contacts between the Ds:Pa base pair and RNAP are observed at both the major- and minor-groove edges. On the major-groove side, the bulky thienyl group of Ds engages in hydrophobic interactions with a loop (spanning residues 564 to 568) of the β subunit, with the closest contact near residue G566 at approximately 4.2 Å (Fig. 2*D*). This interaction provides an additional stabilizing contact that anchors DsTP within the active site.

On the minor-groove side, the minor-groove edge of Ds closely resembles that of adenine and therefore does not introduce UBP-specific interactions, showing only a hydrophobic contact with P427 from the Pro-gate region (residues 425 to 431 of β′) at ~3.9 Å (Fig. 2*D*). In contrast, Pa presents an aldehyde group at its minor-groove edge, which is designed to mimic the 2-keto group of native pyrimidines. This aldehyde group forms hydrophobic interactions with P427 from the Pro-gate and M1273 from the β subunit, with contact distances of approximately 3.8 to 4.1 Å (Fig. 2*D*). These interactions provide a structural basis for polymerase recognition of dPa on template DNA despite its non-canonical base chemistry.

Comparison with an *E. coli* RNAP EC transcribing native substrates [PDB: 6RH3 (21)] shows that the overall position of the Ds:Pa base pair closely overlaps with that of a canonical base pair, with no obvious displacement of the RNA–DNA hybrid (*SI Appendix*, Fig. S6 *A* and *C*). Notably, the aldehyde group of Pa does not perfectly overlap with the 2-keto group of native pyrimidines but is slightly shifted toward the minor-groove edge (*SI Appendix*, Fig. S7 *A*, *B*, *D*, and *E*). Further comparison with a crystal structure of a DNA polymerase incorporating the related Ds:Px base pair [PDB: 5NKL (18)] reveals a similar pairing geometry, with a modest rotation between Pa and Px that likely reflects differences in polymerase environment and the additional chemical modification present in Px (*SI Appendix*, Fig. S7 *A*, *C*, *D*, and *F*).

### Substrate DsTP Loading Induces TL Folding through a Native-Like Recognition Mechanism.

Consistent with activation of the transcriptional machinery, binding of DsTP induces canonical conformational changes associated with catalytically competent complexes, including folding of the TL and bending of the bridge helix (BH; residues 770 to 804 of the β′ subunit) (Fig. 2 *B* and *C*). The resulting folded TL and bent BH stabilize DsTP in a catalytically poised conformation within the active site. Notably, *E. coli* RNAP employs a substrate recognition mechanism for DsTP that closely mirrors the mechanism observed for native NTPs (Fig. 2 *C* and *D* and *SI Appendix*, Fig. S6).

Specifically, base recognition is mediated by TL residue M932 and BH residue T790, which stabilize the Ds and dPa bases, respectively, and support formation of the edge-to-edge base pair (Fig. 2*C* and *SI Appendix*, Fig. S6 *A* and *C*). Ribose recognition follows the canonical pattern, with the 2′- and 3′-hydroxyl groups of the DsTP ribose interacting with β′ residues R425 and N458, respectively (Fig. 2*D* and *SI Appendix*, Fig. S6*B*). Recognition of the triphosphate moiety is likewise conserved: TL residue H936 forms a hydrogen bond with the β-phosphate of DsTP, while the γ-phosphate interacts with β-subunit residues R678 and K1073 (Fig. 2*C* and *SI Appendix*, Fig. S6*A*). Direct comparison with a native substrate-bound RNAP structure (*SI Appendix*, Fig. S6) highlights that these interactions are essentially identical between DsTP and native NTPs.

In addition, the triphosphate moiety of DsTP adopts a chair-like conformation and coordinates two catalytic Mg^2+^ ions through interactions with the β′ P-loop (residues D460 to D464), which contains three conserved aspartate residues (D460, D462, and D464) (Fig. 2*C*). This coordination geometry closely resembles that observed for Ds-containing UBPs and native substrates in DNA polymerase systems, although direct comparison with prior RNAP structures is limited by resolution in earlier studies (*SI Appendix*, Fig. S8).

Together, these observations demonstrate that although Ds:Pa pairing relies on hydrophobic size complementarity rather than canonical hydrogen bonding, it is fully compatible with the conserved substrate-recognition network of *E. coli* RNAP. This compatibility enables Ds:Pa to trigger the same conformational transitions—most notably TL folding—required to promote efficient nucleotide incorporation during transcription.

### Cryo-EM Structure of *E. coli* RNAP EC Containing dDs:PaTP Reveals a Pre-insertion State.

**Fig. 3.**
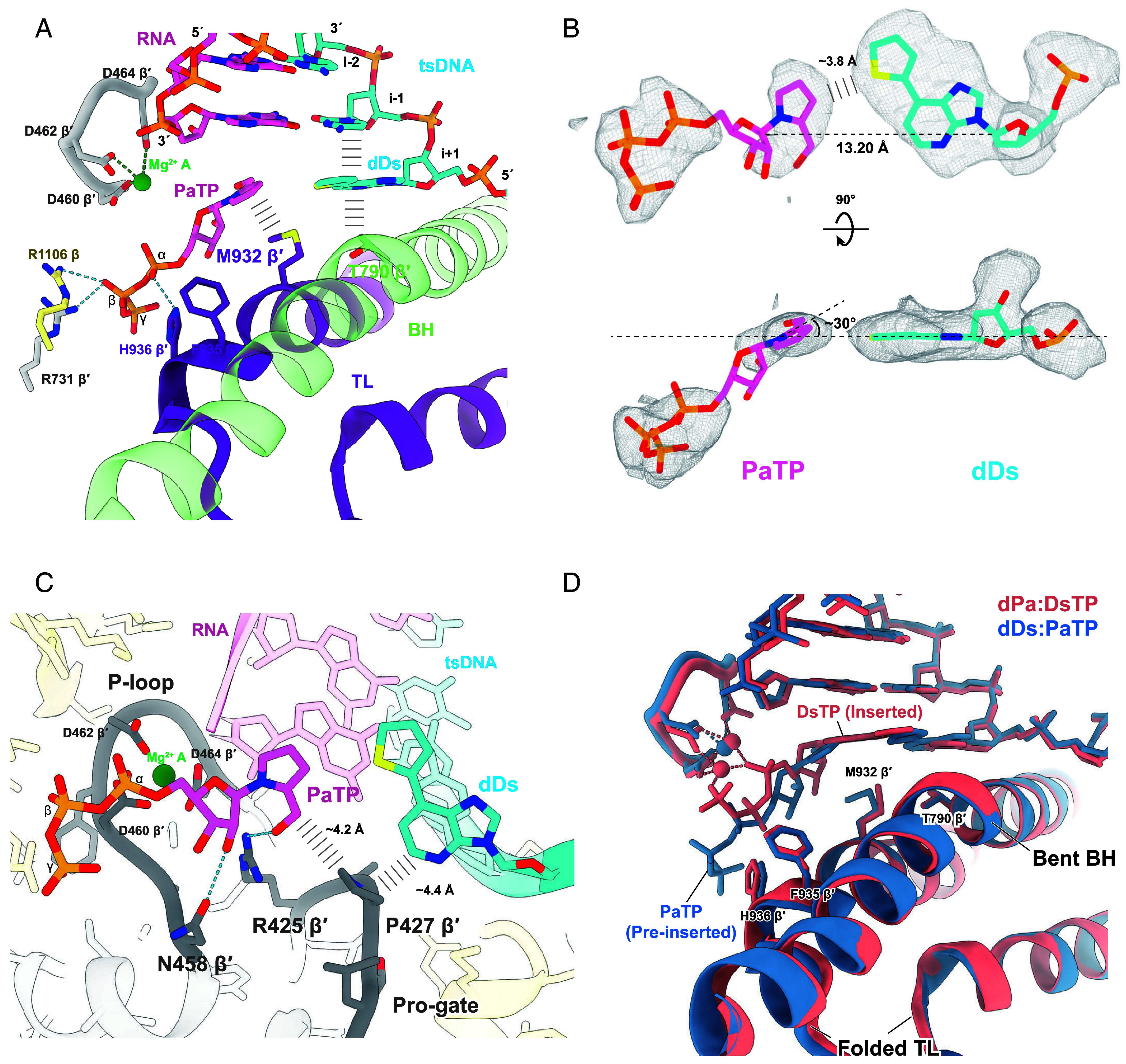
Cryo-EM structure of the *E. coli* RNA polymerase EC containing dDs:PaTP. (*A*) Active-site architecture of the dDs:PaTP EC. Key structural elements include the RNA primer, tsDNA, the β′ P-loop (silver), TL (purple), catalytic Mg^2+^ ions (green), and BH (light green). Residues involved in substrate stabilization are shown as sticks. Hydrogen bonds are depicted as blue dashed lines, and hydrophobic interactions with the Ds:Pa nucleobases are shown as gray bold dashed lines. (*B*) Incomplete base pairing between the incoming PaTP (hot pink) and the templating dDs (cyan). Cryo-EM density is shown as a silver mesh contoured at 6-σ. Hydrophobic contacts are indicated by gray bold dashed lines with distances labeled. The C1′–C1′ distance between the bases is indicated, and the inter-base-plane angle (~30°) is shown in the *Lower* panel. (*C*) Detailed interactions between RNAP and the dDs:PaTP base pair at the major- and minor-groove edges. Hydrophobic interactions with the nucleobase moieties are indicated by gray bold dashed lines with distances labeled. Hydrogen bonds involving the ribose 2′- and 3′-hydroxyl groups are shown as blue dashed lines. (*D*) Structural comparison of two *E. coli* RNAP EC structures containing dPa:DsTP (salmon) and dDs:PaTP (steel blue) from this study.

To further investigate the structural basis for the asymmetric incorporation efficiency of the Ds:Pa base pair, we applied the same reconstitution strategy to assemble an *E. coli* RNAP EC containing a templating dDs at the *i*+1 position paired with the incoming PaTP substrate (Fig. 3 and *SI Appendix*, Fig. S9 and Table S1). From this dataset, we determined a cryo-EM structure at a resolution of 3.24 Å (*SI Appendix*, Fig. S9). This structure captures a distinct intermediate that we term a pre-insertion state, in contrast to the fully inserted state observed for the dPa:DsTP complex, and it is characterized by incomplete base pairing between PaTP and the templating dDs (Fig. 3 *A* and *B*). Despite incomplete substrate insertion, the active site exhibits hallmark conformational features of an activated EC, including a folded TL and a bent BH (Fig. 3*A*).

At the active-site level, the templating dDs base adopts a canonical position, being sandwiched between BH residue T790 and the upstream (*i*–1) nucleobase through stacking interactions (Fig. 3*A*). In contrast, the incoming PaTP is only partially accommodated within the active site and fails to form the intended edge-to-edge, size-complementary base pair with dDs. Instead, the PaTP base is tilted relative to the plane of the templating dDs base, with an inter-base-plane angle of approximately 30°, compared with an angle of ~5° observed for the edge-to-edge base pair in the dPa:DsTP structure (Figs. 2*B* and 3*B*). Consistent with this altered geometry, the C1′–C1′ distance in the pre-insertion state is 13.20 Å, substantially larger than the 10.94 Å distance measured for the fully inserted Ds:Pa base pair (Figs. 2*B* and 3*B*). On the minor-groove side, the Pro-gate residue P427 establishes hydrophobic contacts with both the Ds base and the aldehyde group of Pa, with interaction distances of approximately 4.2 to 4.4 Å (Fig. 3*C*).

### Pre-insertion PaTP Induces TL Folding but Engages a Distinct Substrate-Recognition Network.

Despite incomplete substrate insertion, binding of PaTP in the pre-insertion state still induces TL folding and BH bending. However, the detailed substrate-recognition interactions differ substantially from those observed in the fully inserted DsTP structure (Fig. 3*A*). First, these differences are evident in the recognition of the triphosphate moiety. TL residue H936, which forms a hydrogen bond with the β-phosphate of DsTP in the inserted state, instead interacts with the α-phosphate of PaTP in the pre-insertion state (Figs. 2*C* and 3*A*). Additionally, the γ-phosphate of DsTP interacts with β-subunit residues R678 and K1073 in the inserted state, whereas in the pre-insertion PaTP structure, these interactions are replaced by contacts involving R1106 (β subunit) and R731 (β′ subunit) (Figs. 2*C* and 3*A*). Recognition of the ribose moiety is likewise altered: in the fully inserted state, β′ residues R425 and N458 form hydrogen bonds with the 2′- and 3′-hydroxyl groups of DsTP, respectively, whereas in the pre-insertion state these residues instead interact with the aldehyde group and the 2′-hydroxyl group of PaTP (Figs. 2*D* and 3*C*).

Structural comparison with an *E. coli* RNAP EC containing native substrates shows that both the dPa:DsTP and dDs:PaTP structures adopt TL and BH conformations closely resembling the folded TL and bent BH observed in the catalytically active native complex [PDB: 6RH3 (21)] (Fig. 3*D* and *SI Appendix*, Fig. S6 *C* and *D*). Key residues involved in substrate recognition, including M932, F935, and H936 of the TL and T790 of the BH, occupy nearly identical positions relative to the active site, with only minor rotameric differences in side-chain orientation (Fig. 3*D* and *SI Appendix*, Fig. S6 *C* and *D*). By contrast, an RNAP structure captured in the post-translocation state [PDB: 8FVR (22)] displays an unfolded TL and an unbent BH and serves as a reference for the inactive conformation (Fig. 3*D* and *SI Appendix*, Fig. S6 *E* and *F*).

Taken together, these results indicate that loading of either DsTP or PaTP is sufficient to induce TL folding and promote downstream conformational transitions characteristic of active transcription complexes. However, PaTP populates a distinct pre-insertion state with an alternative interaction network, whereas DsTP achieves a fully inserted, native-like recognition mode. This behavior is likely related to the smaller size (and the resultant lower hydrophobicity) of PaTP relative to the active-site pocket, which permits an alternative binding geometry that is less favorable for complete insertion and may underlie the reduced incorporation efficiency of PaTP.

### A Fully Inserted State Is Required for PaTP Incorporation.

Particle classification analysis indicates that the pre-insertion PaTP-bound complex represents a relatively stable intermediate. In the cryo-EM dataset for the dDs:PaTP complex, only two major classes were recovered: an EC corresponding to the post-translocation state (no PaTP bound) and the PaTP-bound pre-insertion state (*SI Appendix*, Fig. S9*C*). In contrast, too few particles populated a fully inserted PaTP state to permit reliable reconstruction. Together with the interaction network observed for the pre-insertion PaTP, these results suggest that the pre-insertion state constitutes a relatively stable binding mode.

**Fig. 4.**
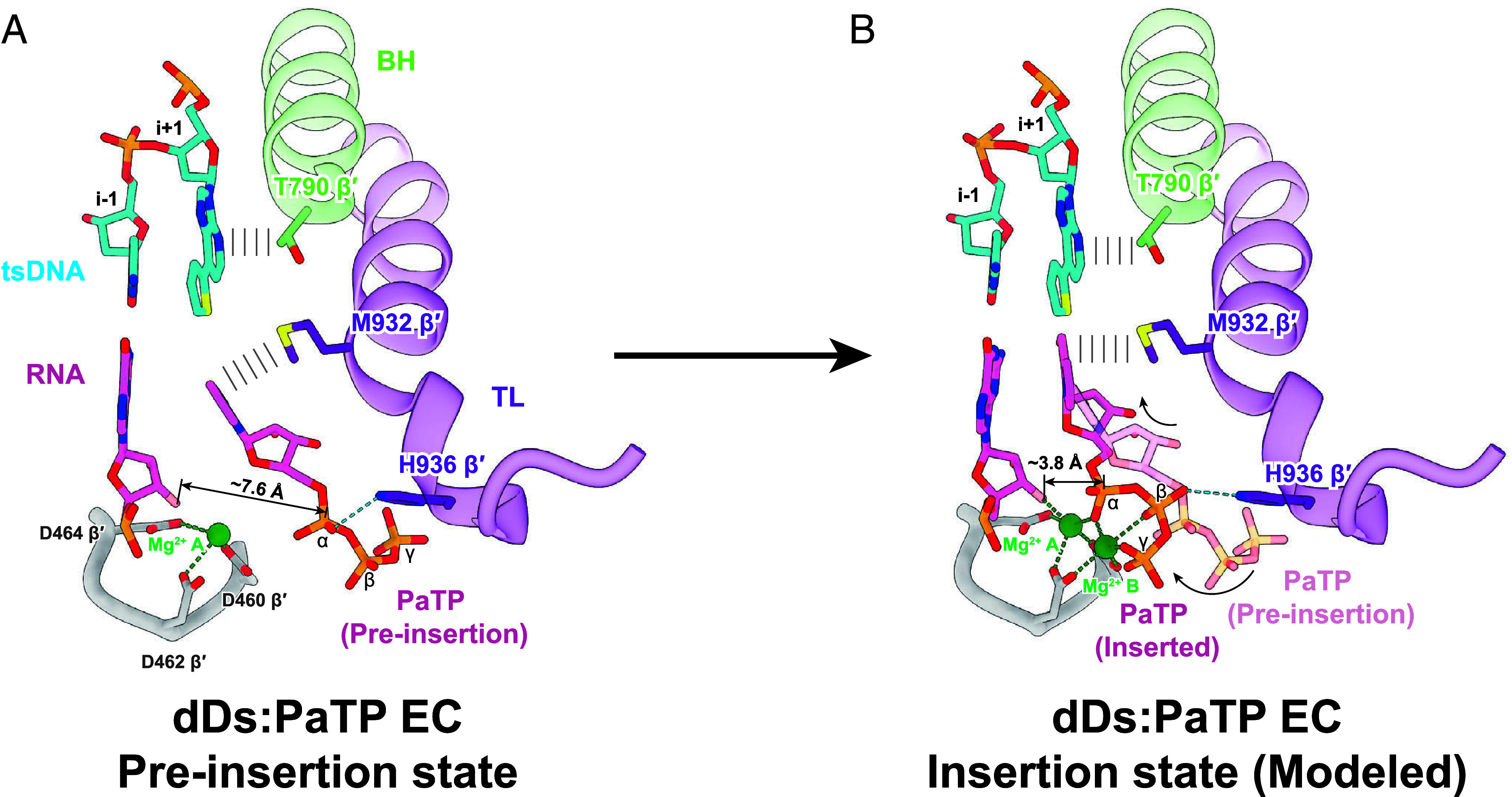
Modeling the insertion state of the dDs:PaTP EC. (*A*) Structure of the dDs:PaTP base pair captured in the pre-insertion state. The distance between the α-phosphate of PaTP and the 3′-hydroxyl group of the RNA primer is indicated. The 3′-hydroxyl of the RNA primer (shown as transparent) was modeled according to standard nucleotide geometry rather than directly resolved in the cryo-EM map. (*B*) Modeled structure of the dDs:PaTP base pair in the insertion state. The preinserted PaTP conformation is superimposed for comparison and shown as a transparent stick model. In the insertion model, the distance between the α-phosphate of PaTP and the 3′-hydroxyl group of the RNA primer is reduced to approximately 3.8 Å, consistent with a catalytically competent geometry. Black arrows indicate the proposed trajectory of PaTP movement from the pre-insertion to the insertion state. In both panels, hydrophobic interactions between the Ds:Pa nucleobases and TL/BH are depicted as gray bold dashed lines, hydrogen bonds are shown as blue dashed lines, and Mg^2+^-mediated coordination is indicated by green dashed lines.

However, the pre-insertion PaTP state is incapable of supporting nucleotide incorporation. First, only a single catalytic metal ion (metal A) is observed in the pre-insertion structure, coordinated by the three conserved aspartate residues in the β′ P-loop, whereas the second metal ion required for catalysis is absent (Figs. 3*A* and 4*A*). To further assess whether this configuration could support phosphodiester bond formation, we modeled the 3′-hydroxyl group of the *i*–1 RNA nucleotide at its expected position based on standard nucleotide geometry. The distance between modeled 3′-hydroxyl group of RNA primer and the α-phosphate of PaTP is approximately 7.6 Å, which is too far to support nucleophilic attack and phosphodiester bond formation (Fig. 4*A*). Therefore, to support catalysis, the PaTP substrate must undergo transition from the pre-insertion state to a fully inserted state.

To further evaluate the requirements for PaTP incorporation, we modeled a fully inserted PaTP-bound *E. coli* RNAP EC based on the experimentally determined inserted DsTP structure (Fig. 4*B*). In this model, PaTP undergoes additional insertion to achieve size-complementary, edge-to-edge pairing with the templating dDs base. Concomitantly, the triphosphate moiety adopts a chair-like conformation that enables coordination of two catalytic Mg^2+^ ions. As a result, the distance between the 3′-hydroxyl group of the *i*–1 RNA nucleotide and the α-phosphate of PaTP is reduced from ~7.6 Å in the pre-insertion state to ~3.8 Å in the fully inserted state, a geometry compatible with catalysis (Fig. 4*B*).

Based on these observations, we propose a two-state loading model for PaTP incorporation, in which PaTP initially binds in a relatively stable pre-insertion state but must transition to a fully inserted state to become catalytically competent. Trapping of PaTP in the pre-insertion state provides a structural explanation for its reduced incorporation efficiency relative to DsTP, which is consistent with the kinetic data.

## Discussion

### A Structural Model for Asymmetric Transcription of Ds:Pa by Multisubunit RNA Polymerase.

**Fig. 5.**
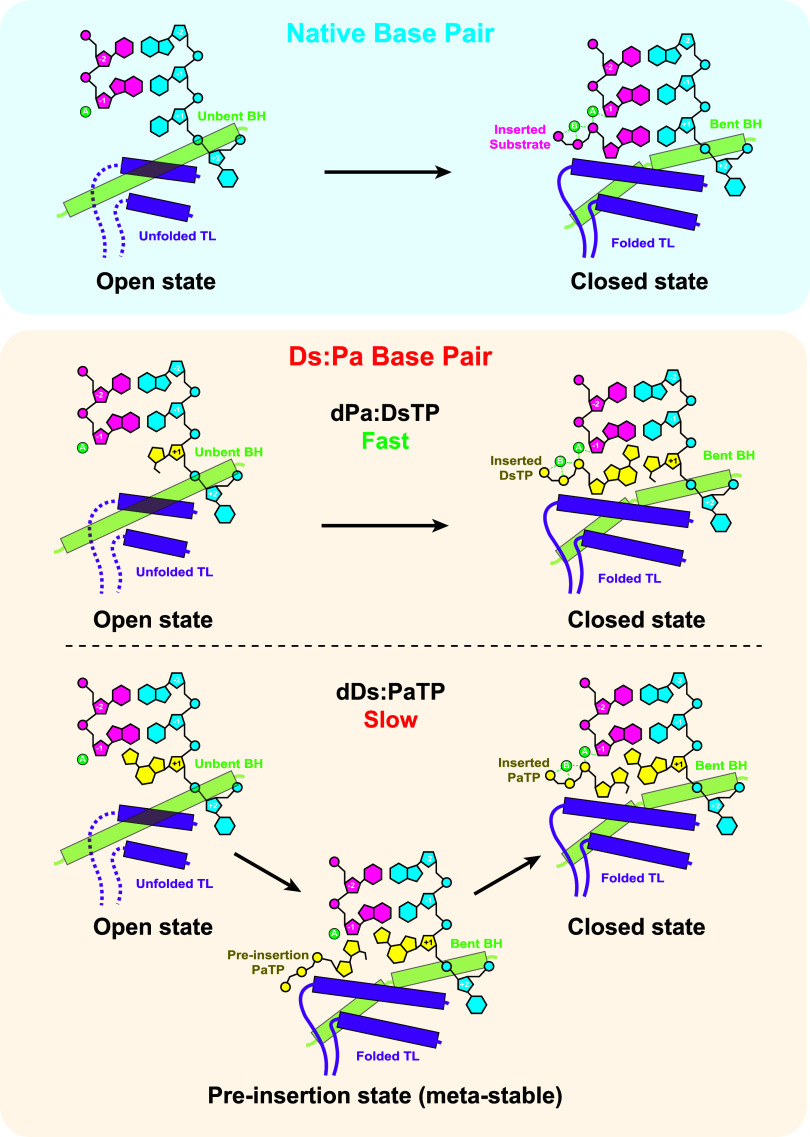
Schematic model for asymmetric transcription of the Ds:Pa base pair by *E. coli* RNA polymerase. The *Upper* panel (light blue background) illustrates native nucleotide incorporation, in which the *E. coli* RNAP transitions from a substrate-unbound state to a fully inserted, catalytically competent state. Loading of the incoming NTP induces TL (purple) folding and BH (light green) bending, thereby organizing the active site for phosphodiester bond formation. The *Lower* panel (light orange background) depicts the asymmetric transcription of the Ds:Pa base pair, accounting for the rapid incorporation of DsTP and the slower incorporation of PaTP. DsTP behaves similarly to a native substrate: upon entry into the active site, it forms an edge-to-edge base pair with the template dPa and subsequently induces TL folding and BH bending, leading to efficient catalysis. In contrast, PaTP is preferentially trapped in a pre-insertion, meta-stable intermediate state in which full base-pair insertion and catalytic geometry are not achieved, but TL adopts closure conformation. Productive incorporation of PaTP requires an additional transition from this preinserted state to a fully inserted state, providing a mechanistic explanation for its reduced incorporation efficiency.

Integrating biochemical, kinetic, and structural data, we propose a mechanistic model for the asymmetric transcription of the Ds:Pa UBP by a multisubunit RNA polymerase, represented here by *E. coli* RNAP (Fig. 5). The hydrophobic Ds:Pa UBP can be efficiently transcribed by *E. coli* RNAP and remains largely orthogonal to native base pairs. Although Ds:Pa relies on a size-complementary, hydrophobic pairing mechanism rather than canonical Watson–Crick hydrogen bonding, its incorporation nonetheless proceeds through a substrate-recognition and catalytic pathway that closely parallels that used for native NTPs. Specifically, loading of the cognate UBP triphosphate enables formation of an edge-to-edge base pair with the complementary templating base, which in turn induces TL folding and BH bending to enclose the active site. These conformational changes promote adoption of a chair-like triphosphate conformation and coordination of two catalytic metal ions, ultimately aligning the α-phosphate of the incoming nucleotide with the 3′-hydroxyl group of the RNA primer in a geometry competent for phosphodiester bond formation.

In contrast to the symmetric incorporation observed for native substrates, Ds:Pa transcription displays pronounced asymmetry depending on strand orientation. DsTP is incorporated more efficiently than PaTP, as reflected by higher k*_pol_max_* and K*_d_app_* values (Fig. 5). Structural analysis reveals that DsTP readily adopts a fully inserted, catalytically competent conformation opposite templating dPa. By comparison, PaTP preferentially populates a pre-insertion state, representing a stable intermediate that must undergo an additional insertion step to achieve full base pairing, proper metal coordination, and productive catalysis. This two-step loading requirement provides a structural explanation for the reduced incorporation efficiency of PaTP.

Together, these findings define the molecular basis for asymmetric Ds:Pa transcription by multisubunit RNAPs and establish design principles for next-generation hydrophobic UBPs. More broadly, this work elucidates key aspects of substrate recognition and activation in RNA polymerase and provides a framework for the rational engineering of UBP-compatible transcription systems.

### Comparison with T7 RNA Polymerase during Ds:Pa Transcription.

The Ds:Pa base pair enables the introduction of Pa substrates conjugated to any functional groups into a specific position in RNA molecules. Using the Ds:Pa base pair, RNA fluorescence labeling, immobilization, and further functionalization via Click chemistry have been explored, and sensing has been pursued using Spinach RNA and Riboswitch methods (13, 14, 16, 17, 23, 24). However, all these reports have employed T7 RNAP, and transcription of Ds:Pa base pairs by other RNA polymerases has not been investigated. Extending Ds:Pa transcription beyond T7 RNAP is therefore a critical step toward broadening its applicability, particularly for recombinant DNA technologies and potential in vivo applications in bacterial systems.

The Ds:Pa unnatural base pair has been systematically investigated in transcription by T7 RNA polymerase, a single-subunit bacteriophage RNAP (19). Similar to our observations with the multisubunit *E. coli* RNAP, Ds:Pa was shown to be efficiently transcribed by T7 RNAP and to exhibit even more pronounced asymmetric incorporation, with DsTP incorporated nearly 540-fold more efficiently than PaTP. However, the structural and mechanistic basis for this asymmetric transcription recognition differs fundamentally between the two transcription systems (19).

A key distinction between multisubunit RNAPs and T7 RNAP lies in the presence of a pre-insertion checkpoint in T7 RNAP during substrate initial selection (19). A side-by-side comparison of the *E. coli* and T7 RNAP active sites (*SI Appendix*, Fig. S10) illustrates this key distinction. The *E. coli* RNAP EC structures determined in this study by cryo-EM capture the active site in a fully inserted state with DsTP (*SI Appendix*, Fig. S10*A*) and in a pre-insertion state with PaTP (*SI Appendix*, Fig. S10*B*). In both structures, the *i*+1 template DNA nucleotide (dPa or dDs) is positioned for base pairing and stacks with the *i*−1 nucleobase, and the TL adopts a closed conformation. By contrast, in the T7 RNAP structures, both the substrate NTP (DsTP or PaTP) and the *i*+1 template DNA nucleotide (dPa or dDs) remain in a pre-insertion arrangement (*SI Appendix*, Fig. S10 *C* and *D*). This difference reflects an additional checkpoint in T7 RNAP that enforces base pairing before the substrate and the *i*+1 template nucleotide are loaded into the active site. This checkpoint is mediated by Y639 on the O-helix, which stacks against the *i*−1 nucleobase of the template strand in the pre-insertion state and is released only once correct base pairing is established at the insertion state, allowing the substrate and the *i*+1 template nucleotide to load into the active site. The capture of this Y639-stacked, pre-insertion conformation in both available T7 RNAP crystal structures of Ds:Pa indicates that it may represent an energetically stable intermediate along the incorporation pathway. This implies that, for T7 RNAP, an additional energy barrier must be overcome to release Y639 and proceed to full substrate insertion, providing a structural basis for the additional checkpoint that Ds:Pa must traverse compared with *E. coli* RNAP.

### Comparison with NaM:TPT3 Incorporation by Multisubunit RNA Polymerases.

NaM:TPT3 represents another well-characterized hydrophobic UBP (20, 25, 26). Structural studies of its analog pair, dNaM:d5SICS, demonstrated formation of an edge-to-edge base pair during DNA polymerase-mediated replication (25). In contrast, during transcription by eukaryotic RNA polymerase II, such an edge-to-edge base-pairing geometry was not observed (20). Instead, the incoming NaMTP substrate was captured at the E site prior to catalysis, while the newly incorporated rNaM nucleotide adopted a distinct “swing” conformation after chemistry, forming a cross-strand intercalated pairing arrangement between the UBPs (20). Notably, TL folding was not detected in these structures, either before or after catalysis, suggesting that the canonical edge-to-edge pairing geometry is energetically disfavored during both pre- and postchemistry states in this system (20).

In contrast, our cryo-EM analyses demonstrate that DsTP can stably load into the active site of *E. coli* RNAP, form an edge-to-edge base pair with templating dPa, and induce TL folding. These observations establish that hydrophobic UBP loading can, in certain contexts, support TL activation and a native-like transcriptional pathway. Together, comparison between Ds:Pa and NaM:TPT3 highlights the mechanistic diversity with which different hydrophobic UBPs are accommodated by multisubunit RNA polymerases and underscores that UBP chemical design plays a decisive role in determining transcriptional outcomes.

## Materials and Methods

### Oligonucleotides and Nucleotides Used in This Study.

RNA oligonucleotides (5′-AUCGAGAGG), 3′-deoxy RNA oligonucleotides (5′-AUCGAGAG/3′dG/), and nontemplate strand (nts) DNA oligonucleotides (5′-TCAGCGAGAGAGAGAAGG) were obtained from Integrated DNA Technologies (IDT). Template strand (ts) DNA oligonucleotides (5′-CCTTCTCTCTCTCGCTGA**X**CCTCTCGATG, where X denotes Ds or Pa) containing unnatural nucleobases were synthesized using an H8 DNA/RNA Synthesizer (K&A Laborgeräte). Standard phosphoramidite chemistry was employed for both natural and unnatural nucleotides, followed by purification via denaturing polyacrylamide gel electrophoresis (4, 27). The unnatural nucleoside triphosphates DsTP and PaTP were prepared according to previously reported procedures (4).

### Purification of *E. coli* RNA Polymerase.

Expression constructs for *E. coli* RNA polymerase, originally developed by the Seth Darst laboratory, were obtained from Addgene, including pEcRNAP6 (Addgene #128940) and pACYCDuet-LEc-rpoZ (Addgene #128837) (28). Recombinant *E. coli* RNA polymerase was expressed and purified using established protocols with minor modifications (29). Briefly, both plasmids were cotransformed into *E. coli* BL21 (DE3) competent cells (Novagen). Cultures were grown to an optical density at 600 nm of approximately 0.6, followed by induction with 0.25 mM IPTG and overnight expression at 20 °C. Cells were harvested by centrifugation and resuspended in Buffer A [50 mM Tris-HCl (pH 7.4), 500 mM NaCl, 5% (v/v) glycerol, 2 mM β-mercaptoethanol, and one tablet of EDTA-free protease inhibitor cocktail (Sigma-Aldrich)]. Cell disruption was carried out using a Microfluidizer, and insoluble debris was removed by high-speed centrifugation. The clarified lysate was applied to Ni-NTA affinity resin, and bound protein was eluted with Buffer A supplemented with 300 mM imidazole. To further enhance sample purity, the eluate was subjected sequentially to heparin affinity chromatography (HiTrap Heparin) and anion-exchange chromatography (HiTrap Q). Fractions corresponding to the intact *E. coli* RNA polymerase holoenzyme (α2β′βω) were pooled and concentrated. During concentration, the buffer was exchanged into storage buffer consisting of 20 mM HEPES (pH 8.0), 150 mM NaCl, 5% (v/v) glycerol, and 1 mM DTT. Purified protein was aliquoted, flash-frozen in liquid nitrogen, and stored at −80 °C until use.

### In Vitro Transcription Assay.

In vitro transcription assays were performed based on previously reported protocols with minor adjustments (29, 30). A miniscaffold was assembled by combining 200 nM ^32^P-labeled RNA, 400 nM template strand DNA (tsDNA), and 600 nM nontemplate strand DNA (ntsDNA) in elongation buffer (EB) containing 20 mM Tris-HCl (pH 7.5), 40 mM KCl, 5 mM DTT, and 5 mM MgCl2. The mixture was annealed by heating to 80 °C for 5 min, followed by gradual cooling to room temperature over a period of at least 2 h.

Elongation complexes were formed by incubating 240 nM *E. coli* RNA polymerase with 40 nM annealed miniscaffold in 1× EB at 30 °C for 20 min. Transcription reactions were initiated by mixing the assembled EC with nucleotide triphosphates, resulting in final reaction conditions of 20 nM miniscaffold and 120 nM RNA polymerase in 1× EB. At the indicated time points, 1.5 μL aliquots were withdrawn and quenched by addition to 6 μL of stop solution containing 90% (v/v) formamide, 50 mM EDTA, 0.05% (w/v) xylene cyanol, and 0.05% (w/v) bromophenol blue. Quenched samples were heat-denatured at 95 °C for 10 min and analyzed by denaturing urea-polyacrylamide gel electrophoresis.

Single-turnover kinetics assays for dPa:DsTP, dPa:ATP, and dDs:PaTP were performed as described above. For each scaffold-substrate configuration, reactions were conducted across a series of substrate concentrations and sampled over a defined time course, with each time-point aliquot resolved by 12% denaturing urea-PAGE. For dPa:DsTP, substrate concentrations from 10 µM to 2 mM and time points from 5 s to 60 min were tested. For dPa:ATP, substrate concentrations from 100 µM to 3 mM and time points from 5 s to 60 min were tested. For dDs:PaTP, substrate concentrations from 75 µM to 2 mM and time points from 5 s to 60 min were tested. Representative gel images are provided in *SI Appendix*, Fig. S2. Band intensities were quantified from gel images using ImageJ (NIH). For each lane, the intensity of the extension product band (+1 or higher) was divided by the sum of all band intensities in that lane to calculate incorporation efficiency (%). To account for the inactive EC fraction, data points within each substrate concentration series were normalized by setting the maximum incorporation value at the reaction plateau to 100%. Incorporation as a function of time was plotted and fitted to a one-phase association nonlinear curve using Prism (version 10) to extract k*_obs_* values. The resulting k*_obs_* values were plotted as a function of substrate concentration and fitted to the Michaelis–Menten equation to obtain k*_pol_max_* and K*_d_app_*. Detailed calculations and uncropped gel images are provided in Dataset S1.

Competition assays were performed based on the in vitro transcription protocol described above. Two substrate pairs were examined for ECs assembled on a dPa template, comprising DsTP/PaTP and DsTP/ATP. For each assay, ECs were incubated with 50 µM of the misincorporated substrate (PaTP or ATP) in the presence of increasing concentrations of the cognate substrate DsTP (2.5, 5, 10, and 20 µM). Single-substrate controls containing 50 µM misincorporated substrate alone or 20 µM cognate DsTP alone were included for comparison. Reactions were sampled at 30 s, resolved by denaturing 20% urea-PAGE, and visualized by ^32^P autoradiography. Results are representative of two independent experiments.

Chase assays were performed based on the in vitro transcription protocol described above. Three scaffold-substrate configurations were tested (*SI Appendix*, Fig. S4): dPa:DsTP, dDs:PaTP, and dT:ATP. For each configuration, the cognate substrate (20 µM DsTP for the dPa template, 200 µM PaTP for the dDs template, or 20 µM ATP for the dT control) was added to the EC and incubated at 30 °C for 5 min to allow single-nucleotide extension at the *i*+1 position. Two downstream treatments were then applied independently to each sample. For the NTP chase, 1 mM each of ATP, GTP, CTP, and UTP was added and the reaction incubated for a further 5 min. For the Mg^2+^ chase, 50 mM Mg^2+^ was added to the incorporation products and incubated for 15 min to assess intrinsic RNA cleavage activity. Four samples were collected for each configuration: the initial EC prior to substrate addition (S1), the complex after UBP incorporation (S2), the complex after NTP chase (S3), and the cleavage product after Mg^2+^ chase (S4). All samples were quenched in stop buffer, denatured at 95 °C for 10 min, and resolved by 12% denaturing urea-PAGE. Results are representative of two independent experiments

### Cryo-EM Sample Preparation.

A miniscaffold composed of 3′-deoxy RNA, tsDNA, and ntsDNA were assembled at a molar ratio of 1.2:1:1.2 by annealing in 1× EB. Elongation complexes were reconstituted by combining *E. coli* RNA polymerase with the annealed miniscaffold at a molar ratio of 1:1.3, followed by incubation at room temperature for 30 min. The final concentration of RNA polymerase in the assembled complexes was approximately 50 μM.

Following complex formation, MgCl2 and CHAPSO were added to final concentrations of 5 mM and 8 mM, respectively. Inclusion of the detergent CHAPSO markedly reduced preferred particle orientation (31). The elongation complexes were then supplemented with 5 mM PaTP or DsTP and incubated at room temperature for an additional 10 min. Samples were subsequently maintained on ice prior to grid preparation.

For grid freezing, 4 μL of the EC containing PaTP or DsTP was applied to freshly plasma-cleaned holey carbon grids (Quantifoil R 1.2/1.3, 2 nm carbon support on copper, 300 mesh; plasma cleaned for 10 s under 100% argon). After a 30 s incubation on the grid surface, excess liquid was removed by manual blotting for 6 s, and the grids were immediately plunge-frozen in liquid ethane. Vitrified grids were stored in liquid nitrogen until cryo-EM data acquisition.

### Cryo-EM Data Collection and Processing.

Cryo-EM data for the dPa:DsTP and dDs:PaTP elongation complexes were collected at the SLAC National Accelerator Laboratory) Cryo-EM Center. Both datasets were acquired on a Titan Krios G3i transmission electron microscope (Thermo Fisher Scientific) operated at 300 keV. The dPa:DsTP dataset was recorded using a Falcon 4i direct electron detector coupled to a Selectris X energy filter, whereas the dDs:PaTP dataset was collected using a K3 direct electron detector equipped with a BioQuantum energy filter. Automated data acquisition was performed using EPU software (32–35). Movies were recorded with calibrated physical pixel sizes of 0.743 Å for the dPa:DsTP dataset and 0.86 Å for the dDs:PaTP dataset. For both datasets, a total electron dose of 30 e^−^/Å^2^ was applied, with images collected over a defocus range of −1.0 to −2.5 μm. In total, 12,618 movies were acquired for the dPa:DsTP dataset and 7,620 movies for the dDs:PaTP dataset. A comprehensive summary of data collection and processing parameters is provided in *SI Appendix*, Table S1.

Image processing for the two datasets followed an identical workflow. Raw movies were imported into CryoSPARC v4.5.3 (36) for motion correction and contrast transfer function (CTF) estimation. Micrographs were manually inspected and filtered to remove low-quality images, including those with CTF fit resolutions poorer than 4.2 Å or relative ice thickness values outside the range of 1.0 to 1.15. Particle picking was performed using the Blob Picker, followed by particle extraction with a box size of 400 pixels and Fourier cropping to bin 4 (100-pixel box). Multiple rounds of 2D classification were conducted, and particles corresponding to well-resolved *E. coli* RNA polymerase views were selected for ab initio model generation and heterogeneous refinement.

Particles from high-quality three-dimensional (3D) classes were subsequently re-extracted at bin 1 using a 400-pixel box size. Additional rounds of heterogeneous refinement were applied to further improve particle homogeneity. To resolve conformational heterogeneity in the TL, local 3D classification was performed using a mask focused on the SI3 domain (highlighted in magenta in *SI Appendix*, Figs. S3*C* and S9*C*), allowing separation of elongation complexes into distinct TL states, including TL-closed and TL-open conformations. Only particles corresponding to the TL-closed state were retained for further analysis, as the TL-open conformation represents a canonical post-translocation state and did not reveal additional mechanistic insights. The selected TL-closed classes were subjected to defocus refinement, global CTF refinement, and nonuniform refinement. Final reconstructions reached overall resolutions of 3.20 Å for the dPa:DsTP complex and 3.24 Å for the dDs:PaTP complex, based on the gold-standard Fourier shell correlation criterion.

Preferred particle orientation was assessed using the Orientation Diagnostics module in cryoSPARC, which implements 3DFSC analysis (37, 38); the conical FSC area ratio (cFAR) and Sampling Compensation Factor (SCF*) were 0.74 and 0.950 for the dPa:DsTP dataset and 0.78 and 0.966 for the dDs:PaTP dataset, both exceeding the recommended thresholds (cFAR > 0.5; SCF* > 0.81), indicating no significant preferred orientation in either dataset (*SI Appendix*, Fig. S11). The corresponding particle orientation distribution sphere shows one (dPa:DsTP) or two (dDs:PaTP) clusters of overrepresented orientations, but particles still populate the rest of the sphere, consistent with the cFAR and SCF* values (*SI Appendix*, Fig. S11).

Atomic model building for both datasets followed the same procedure. A previously published *E. coli* RNA polymerase EC structure [PDB: 6RH3 (21)] was used as the initial model and rigid-body fitted into the cryo-EM density maps using UCSF ChimeraX (39). Coordinate and restraint files for the unnatural nucleotide triphosphates (DsTP and PaTP) and unnatural DNA bases (dPa and dDs) were generated with Phenix (40). These components were manually fitted into the density maps and incorporated into the EC using Coot (41). Final models were obtained through iterative cycles of manual model adjustment in Coot and real-space refinement in Phenix with appropriate geometric restraints. Rotamer restraints were applied with a relaxed weighting (sigma = 3.0) and fitting was performed on residues that were either rotamer outliers or in regions of poor map density (rotamers.fit = outliers_or_poormap), allowing side chains to adopt nonideal rotamer conformations supported by the local density. All structural visualizations were prepared using ChimeraX.

To model the insertion state of dDs:PaTP incorporation, the insertion-state structure of the dPa:DsTP complex was used as the starting template. The template nucleotide dPa and the incoming substrate DsTP were manually replaced with dDs and PaTP, respectively, using Coot. The conformations of dDs and PaTP were subsequently refined under nucleotide geometry restraints and adjusted to satisfy size complementarity for the Ds:Pa UBP and canonical two-metal ion coordination within the active site.

## Supplementary Material

Appendix 01 (PDF)

Dataset S01 (XLSX)

## Data Availability

All source Data are provided with this paper. Cryo-EM density maps and the corresponding atomic coordinates have been deposited in the Electron Microscopy Data Bank (EMDB) and Protein Data Bank (PDB), respectively, under the following accession numbers: dPa:DsTP EC: 3.20 Å cryo-EM map of the *E. coli* RNAP EC containing the dPa:DsTP base pair (EMD-75025) (42) and the corresponding atomic model (PDB: 10AG) (43) and dDs:PaTP EC: 3.24 Å cryo-EM map of the *E. coli* RNAP EC containing the dDs:PaTP base pair (EMD-75030) (44) and the corresponding atomic model (PDB: 10AN) (45). All other data are included in the manuscript and/or supporting information.
