# Supplementary material for: Hydrophobic unnatural base pair promotes trigger loop closure and catalysis in cellular RNA polymerase independent of hydrogen bonding
Source: Proc Natl Acad Sci U S A. 2026 Aug 12;123(33):e2607774123. doi: 10.1073/pnas.2607774123 (PMC13486597; doi:10.1073/pnas.2607774123)
Supplement: Supplementary file 1 [file pnas.2607774123.sapp.pdf]

Supplemental Information

Hydrophobic Unnatural Base Pair Promotes Trigger Loop Closure and Catalysis in Cellular RNA Polymerase Independent of Hydrogen Bonding

Qingrong Li[1,†], Peini Hou[1,†], Michiko Kimoto[2], Juntaek Oh[1,3], Yan Liu[4], Hui Pen Tan[2], Jenny Chong[1], Ichiro Hirao[2*], Dong Wang[1,5,6*]

[1] *Department of Pharmaceutical Sciences, Skaggs School of Pharmacy and Pharmaceutical Sciences, University of California San Diego, La Jolla, CA 92093, USA.*

[2] *Xenolis Pte. Ltd., Singapore 118259, Singapore.*

[3] *Department of Regulatory Science, Graduate School; Institute of Regulatory Innovation through Science; Institute of Integrated Pharmaceutical Sciences. Kyung Hee University, Seoul, Republic of Korea*

[4] *Division of CryoEM and Bioimaging, Stanford Synchrotron Radiation Lightsource, SLAC National Accelerator Laboratory, Stanford University, Menlo Park, CA, USA*

[5] *Department of Molecular and Cellular Medicine, School of Medicine, University of California San Diego, La Jolla, CA 92093, USA.*

[6] *Department of Biochemistry and Molecular Biophysics, University of California San Diego, La Jolla, CA 92093, USA.*

[*]Corresponding authors: ichiro.hirao@xenolis.com, dongwang@ucsd.edu

[†] These authors contribute equally.

Figure Legends

Fig. S1 | Single turn-over nucleotide incorporation kinetic analysis of dPa:DsTP, dPa:ATP, dDs:PaTP.
Kinetic parameters were calculated from biochemical assays with incubation time ranging from 0 to 1 hour depending on the incorporation efficiency. The substrates and their concentrations for each assay are indicated in each corresponding plot. Regression curves were generated using Prism 10.

Fig. S2 | Raw gel data for single turn-over nucleotide incorporation assay.
Raw gel data for Fig. S1. All samples were denatured by heating at 95°C for 10 minutes and subsequently subjected to analysis using a 12% denaturing urea-PAGE gel.

Fig. S3 | Comprehensive specificity and competition analysis of Ds:Pa incorporation by *E. coli* RNA polymerase.
A. Nucleic acid scaffold used for single-nucleotide incorporation assays. The RNA primer (red), template-strand DNA (cyan), and non-template-strand DNA (green) are shown. The template base at the *i*+1 position (X) was varied to include dDs, dPa, dA, dG, dC, and dT.
B. Single-substrate incorporation assays testing the selectivity of *E. coli* RNAP at dDs (upper panel) and dPa (lower panel) templates. Each of six substrates (ATP, GTP, CTP, UTP, DsTP, and PaTP, 100 μM each) was tested individually in separate reactions. Reactions were sampled at 0, 30 s, 5 min, and 30 min. All samples were resolved by denaturing 12% urea-PAGE and visualized by ^{32}P autoradiography. Results are representative of two independent experiments.
C. Single-substrate incorporation assays testing DsTP (upper panel) and PaTP (lower panel) specificity opposite each of the six template bases. *E. coli* RNAP ECs were assembled with the indicated template base at the *i*+1 position. Reactions were initiated by addition of 20 μM DsTP or 200 μM PaTP and sampled at 15 s, 1 min, 5 min, and 30 min. All samples were resolved by denaturing 12% urea-PAGE and visualized by ^{32}P autoradiography. Results are representative of two independent experiments.
D. Competition assay assessing DsTP displacement of PaTP misincorporation at a dPa template. ECs assembled with a dPa template were incubated with 50 μM PaTP in the presence of increasing concentrations of DsTP (2.5, 5, 10, and 20 μM). Single-substrate controls containing 50 μM PaTP alone or 20 μM DsTP alone were included for comparison. Reactions were sampled at 30 s. All samples were resolved by denaturing 20% urea-PAGE and visualized by ^{32}P autoradiography. Results are representative of two independent experiments.
E. Competition assay assessing DsTP displacement of ATP misincorporation at a dPa template. ECs assembled with a dPa template were incubated with 50 μM ATP in the presence of increasing concentrations of DsTP (2.5, 5, 10, and 20 μM). Single-substrate controls containing 50 μM ATP alone or 20 μM DsTP alone were included for comparison. Reactions were sampled at 30 s. All samples were resolved by denaturing 20% urea-PAGE and visualized by ^{32}P autoradiography. Results are representative of two independent experiments. All assays were performed in two independent replicates.

Fig. S4 | NTP chase assay for transcription extension and Mg^{2+}-induced intrinsic cleavage

assays following Ds:Pa incorporation in both orientations.
A. Schematic overview of experimental design. *Ec*RNAP ECs were assembled with dPa, dDs, or dT positioned at the i+1 template site. Lane S1: transcript products from assembled *Ec*RNAP ECs without substrate addition and served as a control (9mer). Lane S2: transcript products from *Ec*RNAP ECs with the cognate nucleotide triphosphate (20 μM DsTP for the dPa template, 200 μM PaTP for the dDs template, or 20 μM ATP for the dT control) to achieve single-nucleotide extension (5 min incubation). Lane S3: chase reaction was prepared by adding 1 mM each of the four natural NTPs mixture (ATP, GTP, CTP, and UTP) to the extended S2 complex for additional 5 min to evaluate translocation and resumed elongation. Lane S4: Intrinsic cleavage reaction was prepared by adding 50 mM $MgCl_2$ to the extended S2 complex for 15 min to assess intrinsic RNA cleavage activity. All samples were resolved by denaturing 12% urea-PAGE and visualized by ^{32}P autoradiography.
B. Representative gel images of the NTP chase and Mg^{2+}-induced cleavage assays. Sample preparations are as described in panel A. Results are representative of two independent experiments.

Fig. S5 | Cryo-EM data processing of the *E. coli* RNA polymerase elongation complex containing dPa:DsTP.
A. Representative raw micrographs of the *E. coli* RNAP EC containing dPa:DsTP.
B. Reference-free 2D class averages of the *E. coli* RNAP EC containing dPa:DsTP.
C. Cryo-EM data processing workflow for the *E. coli* RNAP EC. The SI3 domain is highlighted in magenta. Closed-up views of the active site are shown for substrate-bound and substrate-unbound classes. Cryo-EM density is displayed as a silver volume.
D. Final cryo-EM map of the *E. coli* RNAP EC, colored by local resolution. The map is displayed in a cross-sectional view through the active site to reveal internal structural features.
E. Gold-standard Fourier shell correlation (FSC) curves for the masked core region of the *E. coli* RNAP EC reconstruction.

Fig. S6 | Structural comparison with native *E. coli* RNA polymerase elongation complexes.
A. Active-site architecture of the native dG:CTP elongation complex (PDB: 6RH3[19]). Key structural elements include the RNA primer, template-strand DNA (tsDNA), the β′ P-loop (silver), TL (purple), catalytic Mg^{2+} ions (green), and the BH (light green). Residues involved in substrate stabilization are shown as sticks. Hydrogen bonds are depicted as blue dashed lines, and interactions with the G:C nucleobases are indicated by gray bold dashed lines.
B. Detailed interactions between RNAP and the native dG:CTP base pair at the major- and minor-groove edges (PDB: 6RH3). Hydrogen bonds involving the ribose 2′- and 3′-hydroxyl groups are shown as blue dashed lines.
C–F. Structural comparison of active site conformations among four *E. coli* RNA polymerase elongation complexes containing a native substrate (dG:CTP; PDB: 6RH3, light green), UBP substrates (dPa:DsTP, salmon; dDs:PaTP, steel blue), or no bound substrate (PDB: 8FVR[20], gray). Panels C–F show pairwise structural comparisons: dPa:DsTP versus dG:CTP (C), dDs:PaTP versus dG:CTP (D), dPa:DsTP versus the substrate-free complex (E), and dDs:PaTP versus the substrate-free complex (F).

Fig. S7 | Structural comparison of native and unnatural base pairing across RNA and DNA polymerases.

A–C. Chemical structures of base pairs, including the Ds:Pa base pair (panel A, this study), the native G:C base pair (panel B, *E. coli* RNAP; PDB: 6RH3[19]), and the UBP Ds:Px (panel C, DNA polymerase; PDB: 5NKL[16]).
D. Structure of the Ds:Pa base pair observed in the dPa:DsTP elongation complex determined in this study.
E, F. Structural superposition of the Ds:Pa base pair with the native G:C base pair in RNAP (penal E, light green) and with the UBP Ds:Px base pair in DNA polymerase (penal F, purple).

Fig. S8 | Structural comparison between UBP-containing *E. coli* RNAP EC and DNA polymerases in two-metal-ion coordination.
A. Two-metal-ion coordination in the *E. coli* RNAP EC containing dPa:DsTP at the i+1 position.
B, C. Two-metal-ion coordination in DNA polymerase with UBP substrate dPxTP (panel B, PDB: 5NKL[16]) and native substrate dCTP (panel C, PDB: 3KTQ[39]) .
In all panels, key elements are colored as follows: Mg^{2+} ions (green), RNA (hot pink), DNA (cyan), incoming substrate NTP/dNTP (gold), and water molecules (W, red sphere). Coordination bonds to Mg^{2+} are indicated by green dashed lines. The labels "α," "β," and "γ" denote the positions of the phosphate groups of the incoming nucleotide. The chair-like conformation of the triphosphate moiety is indicated by black labels.

Fig. S9 | Cryo-EM data processing of the *E. coli* RNA polymerase elongation complex containing dDs:PaTP.
A. Representative raw micrographs of the *E. coli* RNAP EC containing dDs:PaTP.
B. Reference-free 2D class averages of the *E. coli* RNAP EC containing dDs:PaTP.
C. Cryo-EM data processing workflow for the *E. coli* RNAP EC. The SI3 domain is highlighted in magenta. Closed-up views of the active site are shown for substrate-bound and substrate-unbound classes. Cryo-EM density is displayed as a silver volume.
D. Final cryo-EM map of the *E. coli* RNAP EC, colored by local resolution. The map is displayed in a cross-sectional view through the active site to reveal internal structural features.
E. Gold-standard Fourier shell correlation (FSC) curves for the masked core region of the *E. coli* RNAP EC reconstruction.

Fig. S10 | Structural comparison of the active site between *E. coli* and T7 RNAP transcribing Ds:Pa
A, B. Active site architecture of *E. coli* RNAP transcribing dPa:DsTP (A, salmon) and dDs:PaTP (B, steel blue), determined in this study by cryo-EM. The active site comprises the template DNA (tsDNA), RNA primer, trigger loop (TL), bridge helix (BH), and the incoming nucleotide triphosphate. Side chains of T790 and M932 are shown as sticks, and hydrophobic interactions including base stacking are indicated by gray dashed lines.
C, D. Active site architecture of T7 RNAP transcribing dPa:DsTP (C, gold, PDB 8DH4) and dDs:PaTP (D, hot pink, PDB 8DH1), from previously reported crystal structures. The active site comprises the template DNA (tsDNA), RNA primer, O-helix, and the incoming nucleotide triphosphate. The side chain of Y639 is shown as a stick and highlighted in green, and hydrophobic interactions including base stacking are indicated by gray dashed lines.
E, F. Superposition of the *E. coli* and T7 RNAP active sites containing dPa:DsTP (E) and dDs:PaTP (F).

Fig. S11 | 3DFSC analysis of preferred particle orientation for the dPa:DsTP and dDs:PaTP datasets

A, B. 3DFSC analysis of the dPa:DsTP (A) and dDs:PaTP (B) datasets. Each of the 3,072 conical FSC (cFSC) curve represents the directional Fourier shell correlation along a uniformly sampled viewing direction. The dark line is the mean cFSC; the shaded regions show the ±1 s.d. range and the min-max envelope. The green histogram shows the distribution of resolutions at which individual cFSC curves cross the 0.143 threshold. cFAR and SCF* values, given in each panel title, exceed the thresholds of 0.5 and 0.81 (cFAR = 0.74/0.78, SCF* = 0.950/0.966 for A/B), indicating the absence of significant preferred orientation.

C, D. Particle orientation distribution sphere overlaid on the cryo-EM map for the dPa:DsTP (C) and dDs:PaTP (D) datasets. Each spike points along one viewing direction, with spike length and color encoding the number of particle images assigned to that direction (red/long = many, blue/short = few).

Fig. S1

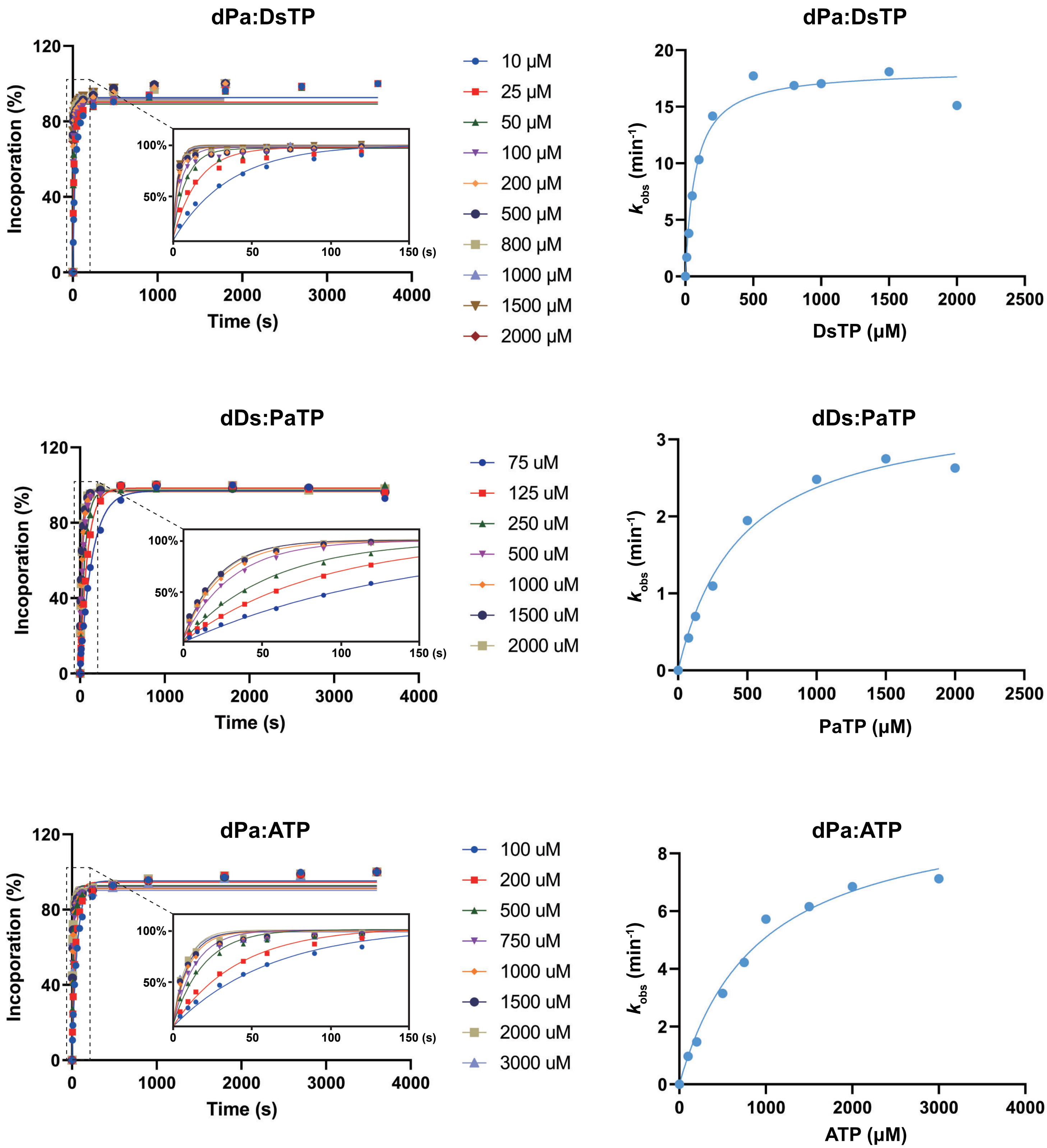
dPa:DsTP
Incoporation (%)
Time (s)
10 μM
25 μM
50 μM
100 μM
200 μM
500 μM
800 μM
1000 μM
1500 μM
2000 μM
dPa:DsTP
kobs (min-1)
DsTP (μM)
dDs:PaTP
Incoporation (%)
Time (s)
75 uM
125 uM
250 uM
500 uM
1000 uM
1500 uM
2000 uM
dDs:PaTP
kobs (min-1)
PaTP (μM)
dPa:ATP
Incoporation (%)
Time (s)
100 uM
200 uM
500 uM
750 uM
1000 uM
1500 uM
2000 uM
3000 uM
dPa:ATP
kobs (min-1)
ATP (μM)

Fig. S2

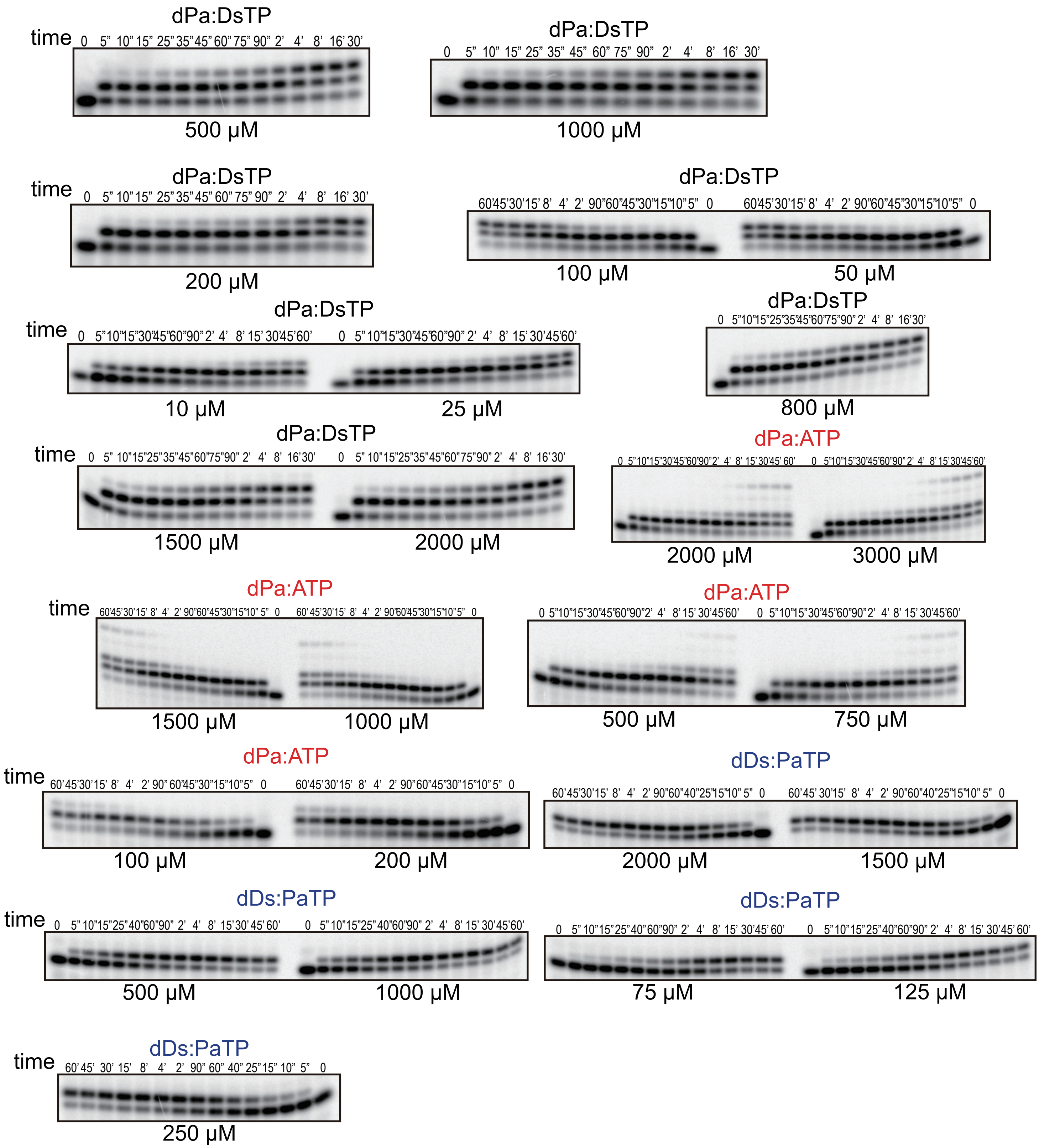

dPa:DsTP
time
500 μM
dPa:DsTP
1000 μM
dPa:DsTP
time
200 μM
dPa:DsTP
100 μM
50 μM
dPa:DsTP
time
10 μM
25 μM
dPa:DsTP
800 μM
dPa:DsTP
time
1500 μM
2000 μM
dPa:ATP
2000 μM
3000 μM
dPa:ATP
time
1500 μM
1000 μM
dPa:ATP
500 μM
750 μM
dPa:ATP
time
100 μM
200 μM
dDs:PaTP
2000 μM
1500 μM
dDs:PaTP
time
500 μM
1000 μM
dDs:PaTP
75 μM
125 μM
dDs:PaTP
time
250 μM

Fig. S3

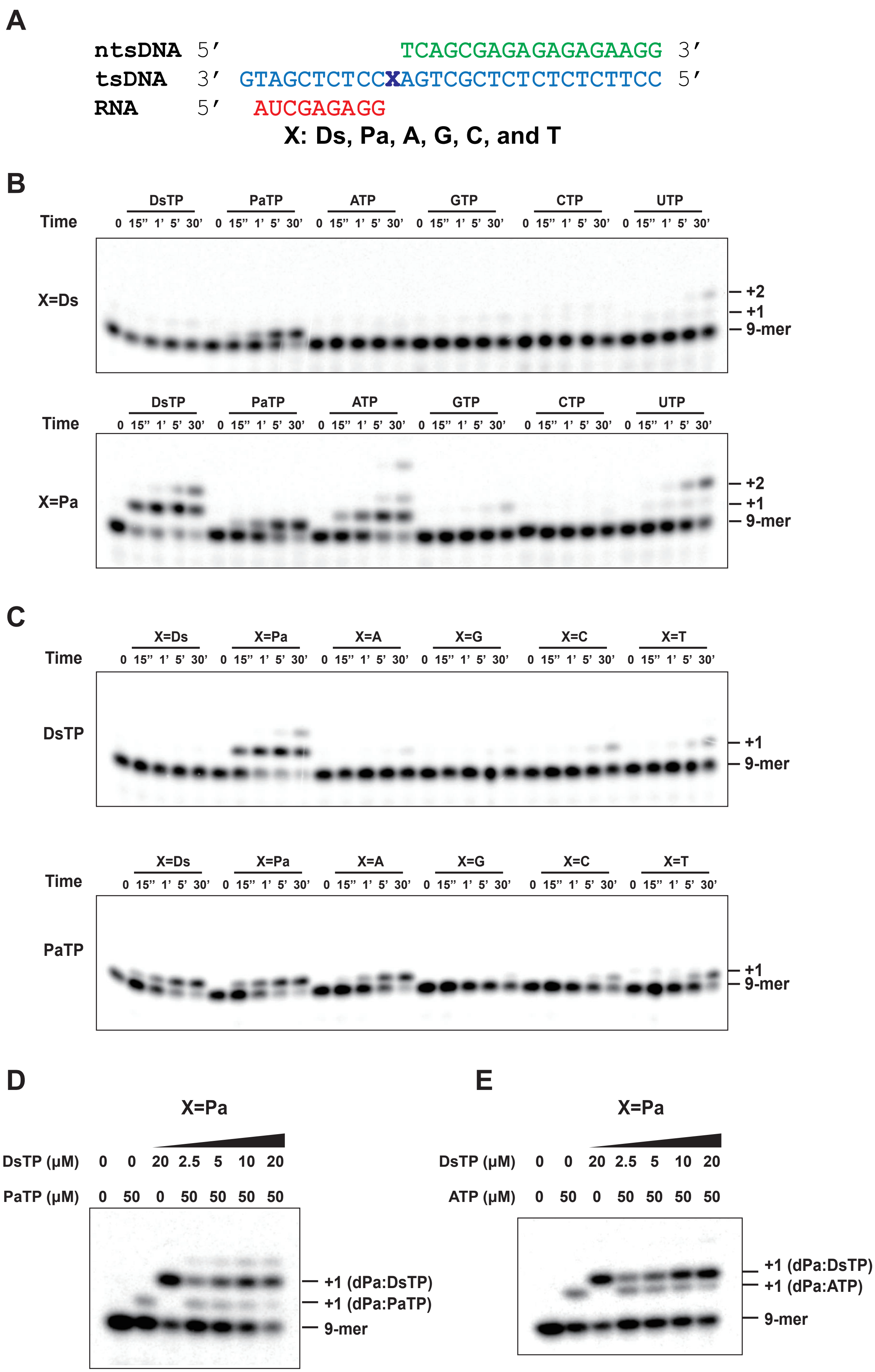
A
ntsDNA 5′ TCAGCGAGAGAGAGAAGG 3′
tsDNA 3′ GTAGCTCTCCXAGTCGCTCTCTCTCTTCC 5′
RNA 5′ AUCGAGAGG
X: Ds, Pa, A, G, C, and T
B
DsTP PaTP ATP GTP CTP UTP
Time 0 15" 1' 5' 30'
X=Ds
+2
+1
9-mer
X=Pa
C
X=Ds X=Pa X=A X=G X=C X=T
DsTP
PaTP
D
X=Pa
DsTP (μM) 0 0 20 2.5 5 10 20
PaTP (μM) 0 50 0 50 50 50 50
+1 (dPa:DsTP)
+1 (dPa:PaTP)
9-mer
E
X=Pa
DsTP (μM) 0 0 20 2.5 5 10 20
ATP (μM) 0 50 0 50 50 50 50
+1 (dPa:DsTP)
+1 (dPa:ATP)
9-mer

Fig. S4

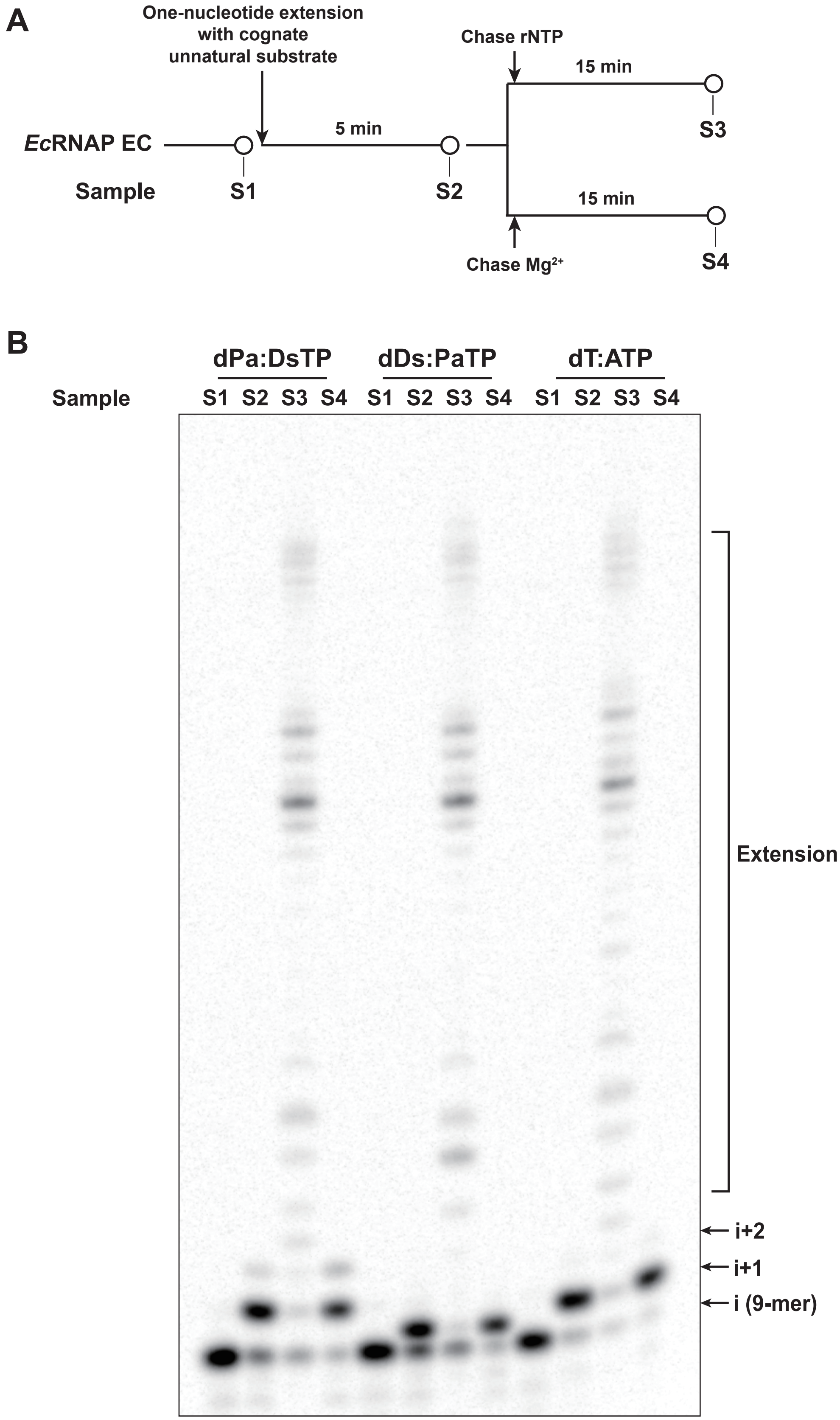
A
One-nucleotide extension
with cognate
unnatural substrate
Chase rNTP
15 min
S3
EcRNAP EC
5 min
Sample
S1
S2
15 min
S4
Chase Mg2+
B
dPa:DsTP
dDs:PaTP
dT:ATP
Sample
S1 S2 S3 S4 S1 S2 S3 S4 S1 S2 S3 S4
Extension
i+2
i+1
i (9-mer)

Fig. S5

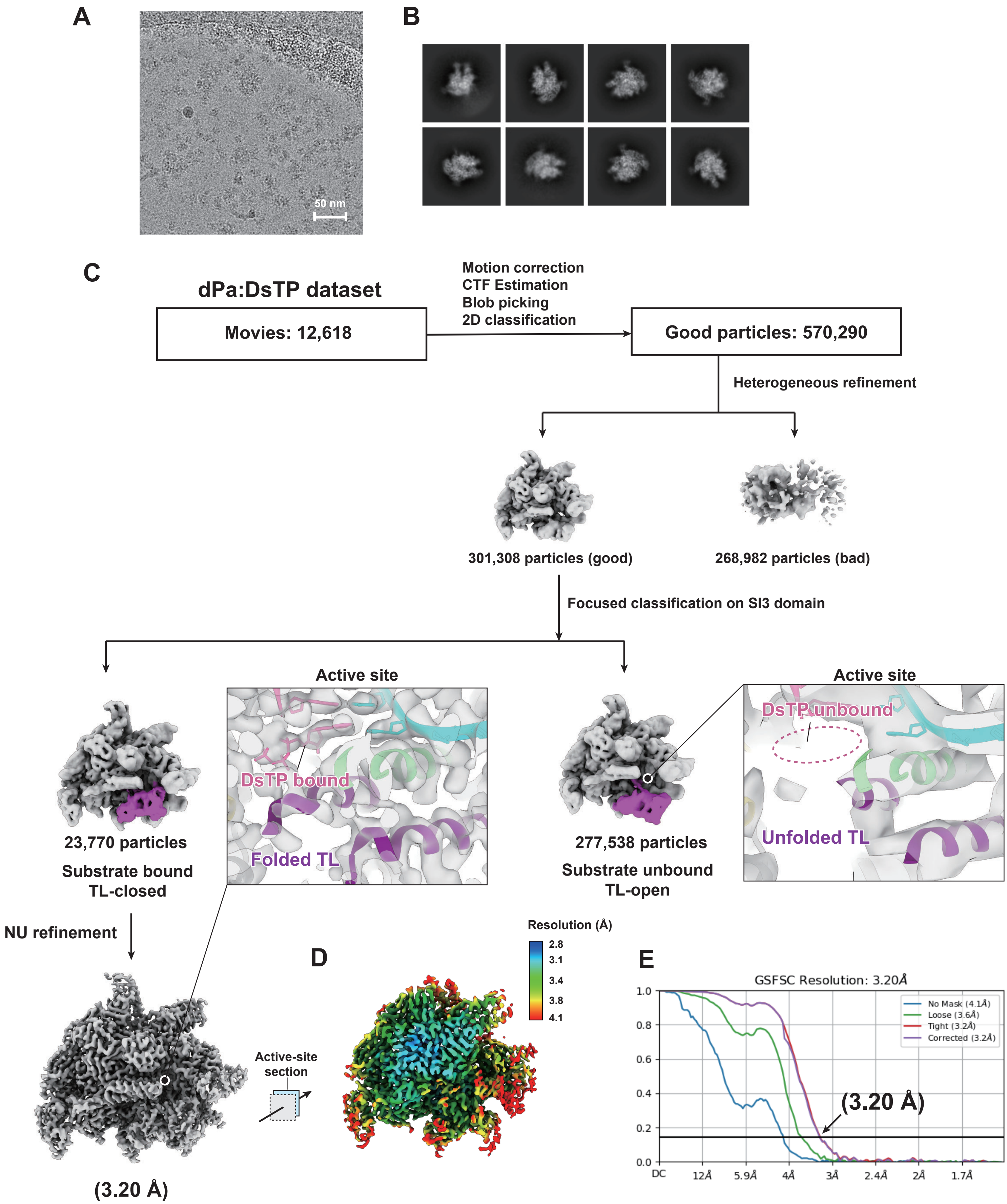

A
50 nm
B
C
dPa:DsTP dataset
Movies: 12,618
Motion correction
CTF Estimation
Blob picking
2D classification
Good particles: 570,290
Heterogeneous refinement
301,308 particles (good)
268,982 particles (bad)
Focused classification on SI3 domain
Active site
DsTP bound
Folded TL
23,770 particles
Substrate bound
TL-closed
Active site
DsTP unbound
Unfolded TL
277,538 particles
Substrate unbound
TL-open
NU refinement
(3.20 Å)
Active-site
section
D
Resolution (Å)
2.8
3.1
3.4
3.8
4.1
E
GSFSC Resolution: 3.20Å
No Mask (4.1Å)
Loose (3.6Å)
Tight (3.2Å)
Corrected (3.2Å)
(3.20 Å)
1.0
0.8
0.6
0.4
0.2
0.0
DC
12Å
5.9Å
4Å
3Å
2.4Å
2Å
1.7Å

Fig. S6

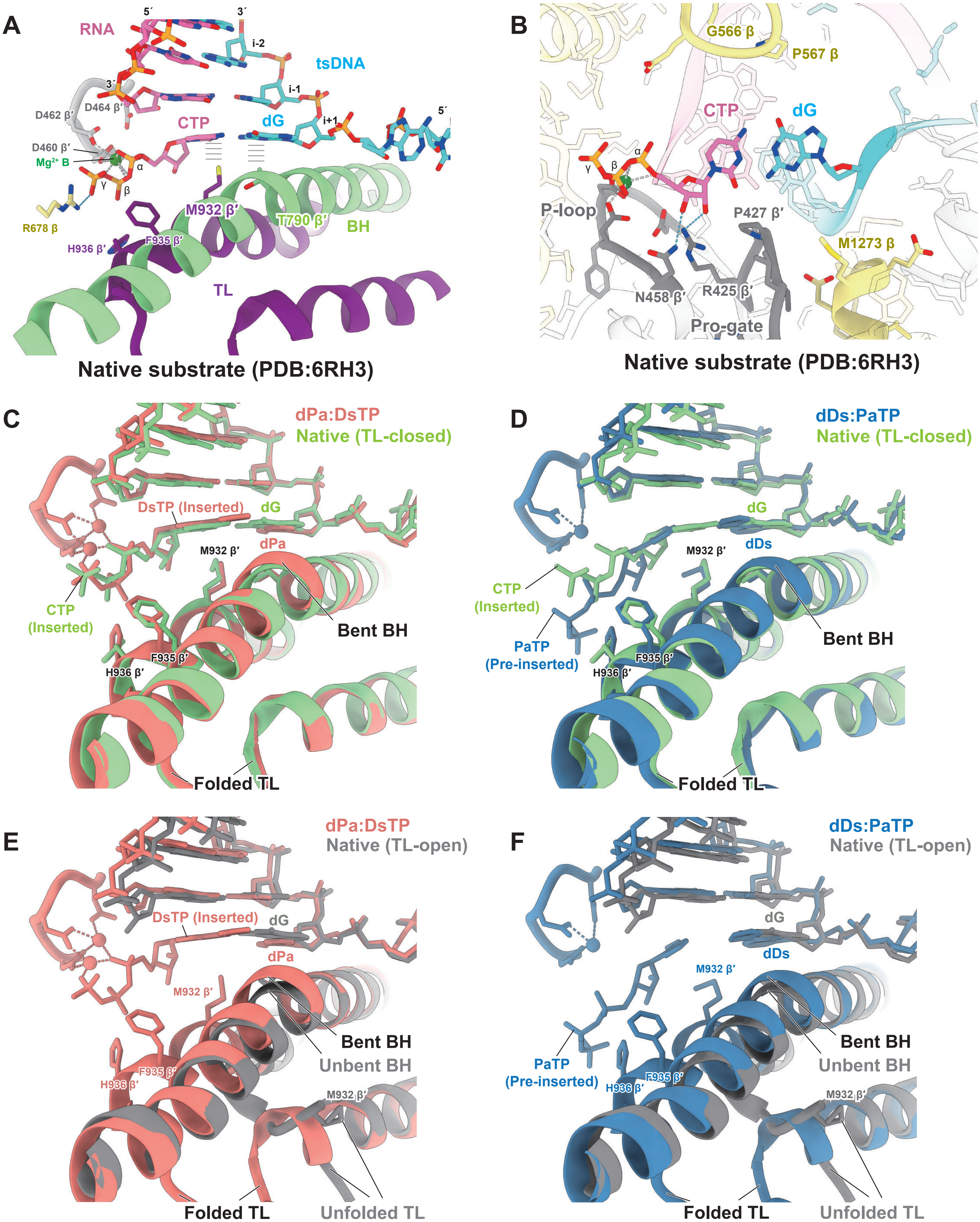
A
5´
3´
RNA
i-2
tsDNA
3´
i-1
D462 β′
D464 β′
CTP
dG
i+1
5´
D460 β′
Mg²⁺ B
α
γ
β
R678 β
M932 β′
T790 β′
BH
H936 β′
F935 β′
TL
Native substrate (PDB:6RH3)
B
G566 β
P567 β
CTP
dG
γ
β
α
P-loop
P427 β′
M1273 β
N458 β′
R425 β′
Pro-gate
Native substrate (PDB:6RH3)
C
dPa:DsTP
Native (TL-closed)
DsTP (Inserted)
dG
M932 β′
dPa
CTP
(Inserted)
Bent BH
H936 β′
F935 β′
Folded TL
D
dDs:PaTP
Native (TL-closed)
dG
M932 β′
dDs
CTP
(Inserted)
PaTP
(Pre-inserted)
Bent BH
F935 β′
H936 β′
Folded TL
E
dPa:DsTP
Native (TL-open)
DsTP (Inserted)
dG
dPa
M932 β′
Bent BH
Unbent BH
H936 β′
F935 β′
M932 β′
Folded TL
Unfolded TL
F
dDs:PaTP
Native (TL-open)
dG
dDs
M932 β′
Bent BH
Unbent BH
PaTP
(Pre-inserted)
H936 β′
F935 β′
M932 β′
Folded TL
Unfolded TL

Fig. S7

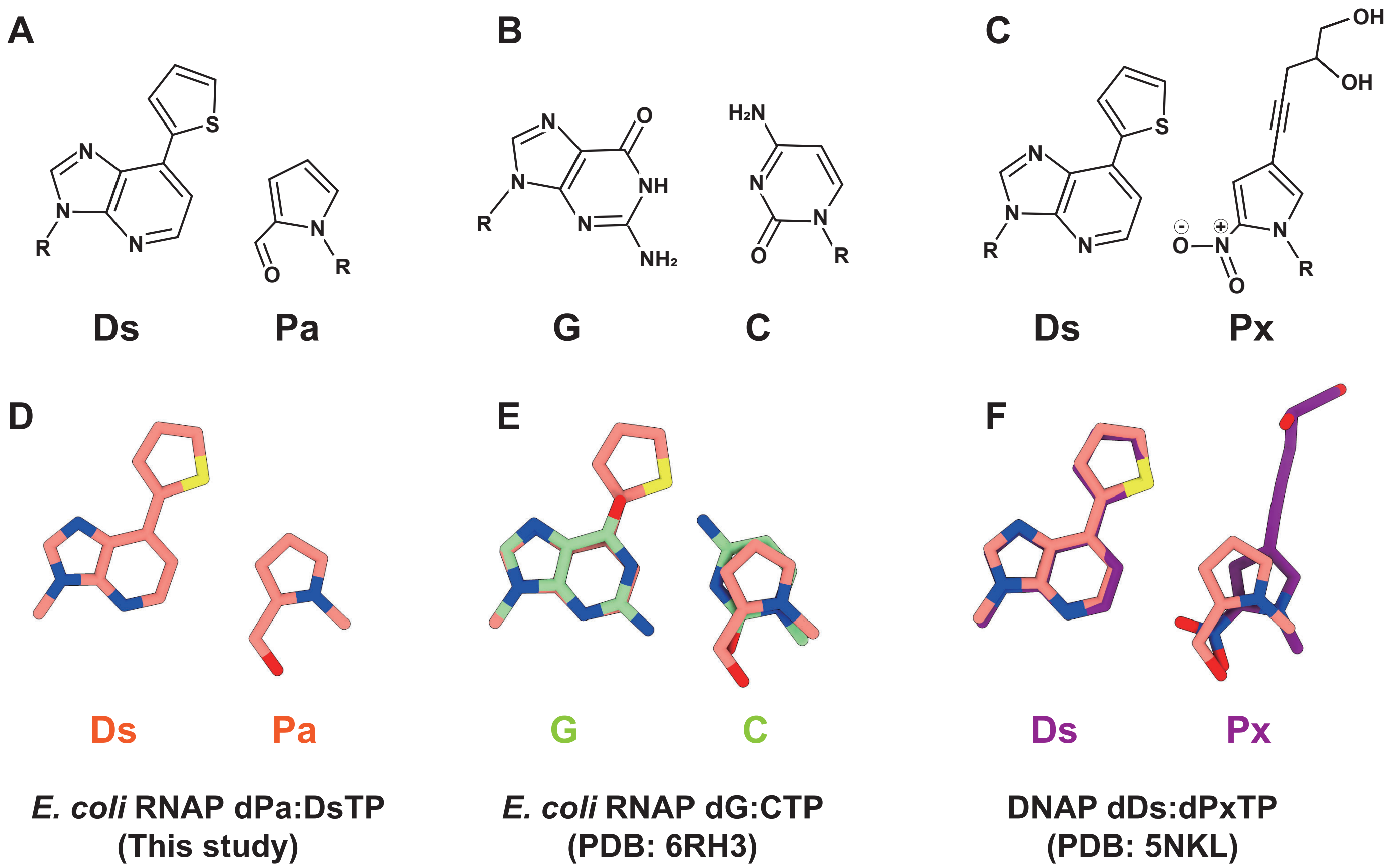

A
B
C
OH
OH
H₂N
O
N
S
NH
R
N
O
NH₂
Ds
Pa
G
C
Ds
Px
D
E
F
Ds
Pa
G
C
Ds
Px
E. coli RNAP dPa:DsTP
(This study)
E. coli RNAP dG:CTP
(PDB: 6RH3)
DNAP dDs:dPxTP
(PDB: 5NKL)

Fig. S8

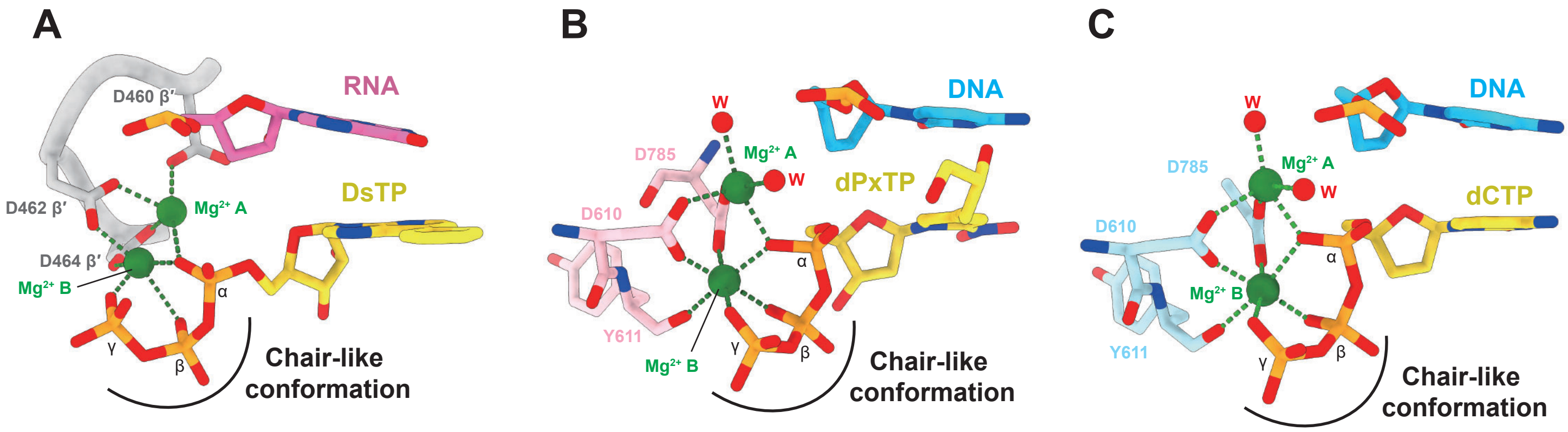

E. coli RNAP dPa:DsTP (This study)

DNAP dDs:dPxTP (PDB: 5NKL)

DNAP dG:dCTP (PDB: 3KTQ)

Fig. S9

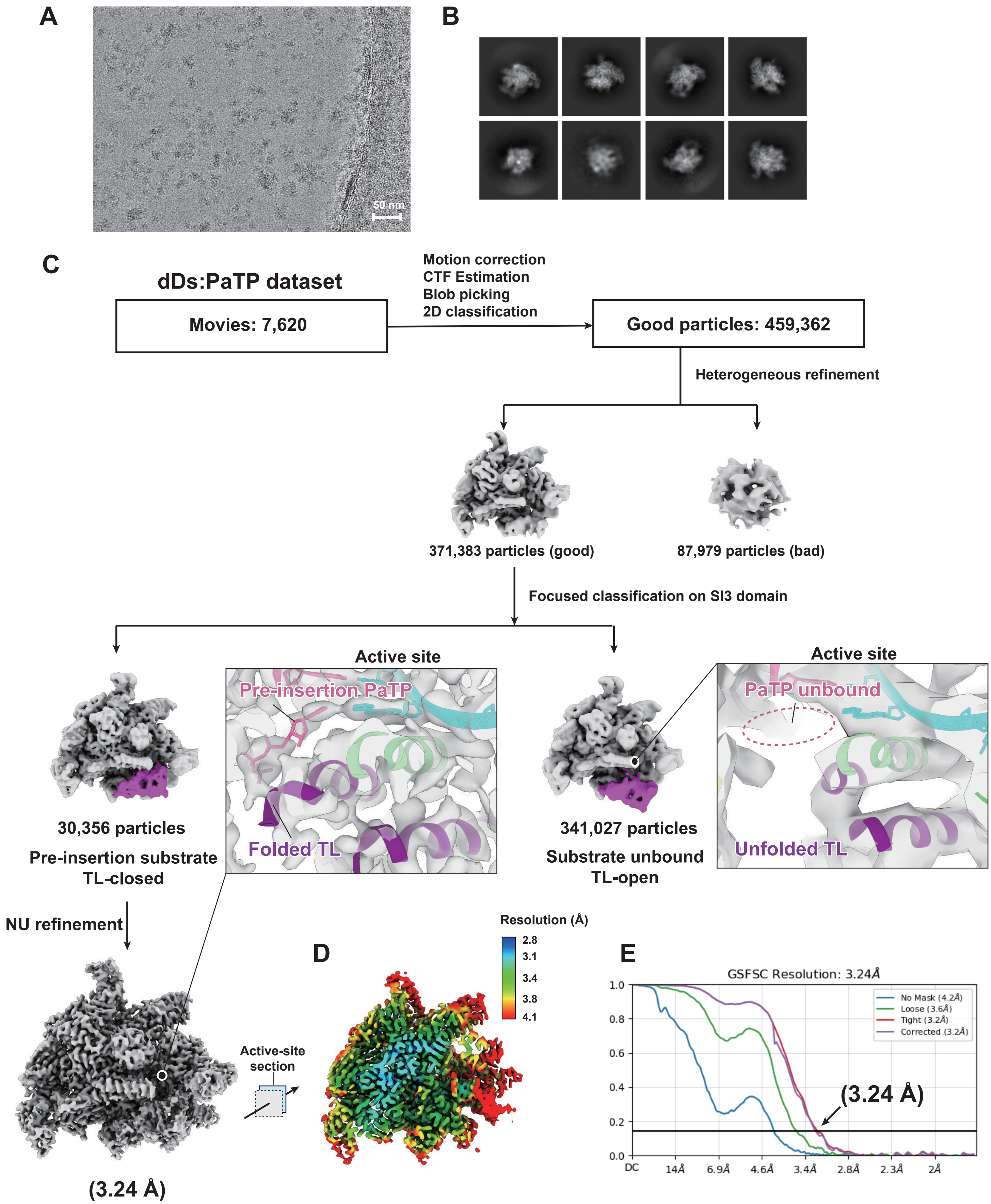
A
50 nm
B
C
dDs:PaTP dataset
Movies: 7,620
Motion correction
CTF Estimation
Blob picking
2D classification
Good particles: 459,362
Heterogeneous refinement
371,383 particles (good)
87,979 particles (bad)
Focused classification on SI3 domain
Active site
Pre-insertion PaTP
Folded TL
30,356 particles
Pre-insertion substrate
TL-closed
Active site
PaTP unbound
Unfolded TL
341,027 particles
Substrate unbound
TL-open
NU refinement
(3.24 Å)
Active-site
section
D
Resolution (Å)
2.8
3.1
3.4
3.8
4.1
E
GSFSC Resolution: 3.24Å
No Mask (4.2Å)
Loose (3.6Å)
Tight (3.2Å)
Corrected (3.2Å)
(3.24 Å)
1.0
0.8
0.6
0.4
0.2
0.0
DC
14Å
6.9Å
4.6Å
3.4Å
2.8Å
2.3Å
2Å

Fig. S10

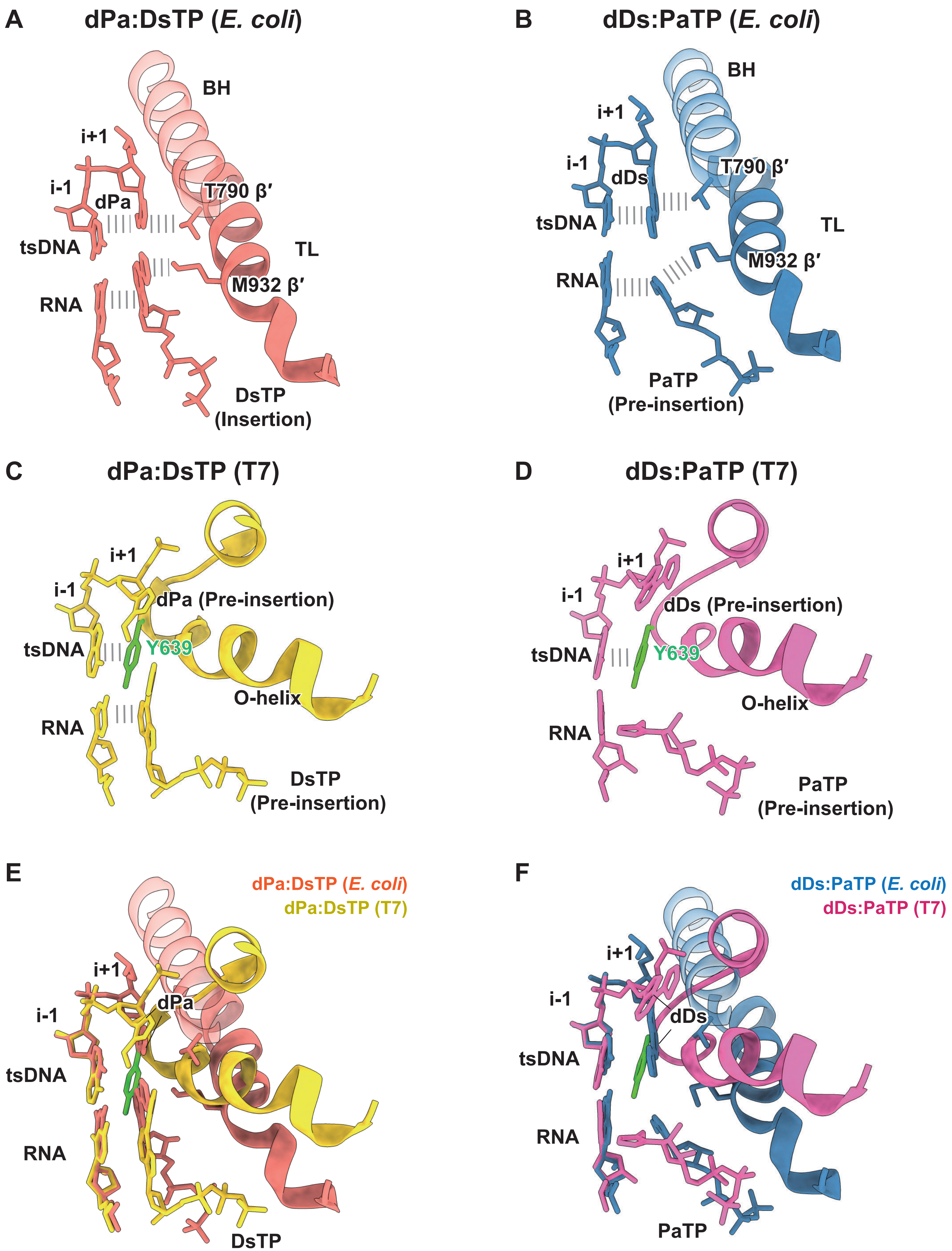

A
dPa:DsTP (E. coli)
BH
i+1
i-1
dPa
T790 β′
tsDNA
TL
M932 β′
RNA
DsTP
(Insertion)
B
dDs:PaTP (E. coli)
BH
i+1
i-1
dDs
T790 β′
tsDNA
TL
M932 β′
RNA
PaTP
(Pre-insertion)
C
dPa:DsTP (T7)
i+1
i-1
dPa (Pre-insertion)
tsDNA
Y639
O-helix
RNA
DsTP
(Pre-insertion)
D
dDs:PaTP (T7)
i+1
i-1
dDs (Pre-insertion)
tsDNA
Y639
O-helix
RNA
PaTP
(Pre-insertion)
E
dPa:DsTP (E. coli)
dPa:DsTP (T7)
i+1
i-1
dPa
tsDNA
RNA
DsTP
F
dDs:PaTP (E. coli)
dDs:PaTP (T7)
i+1
i-1
dDs
tsDNA
RNA
PaTP

Fig. S11

A **dPa:DsTP**

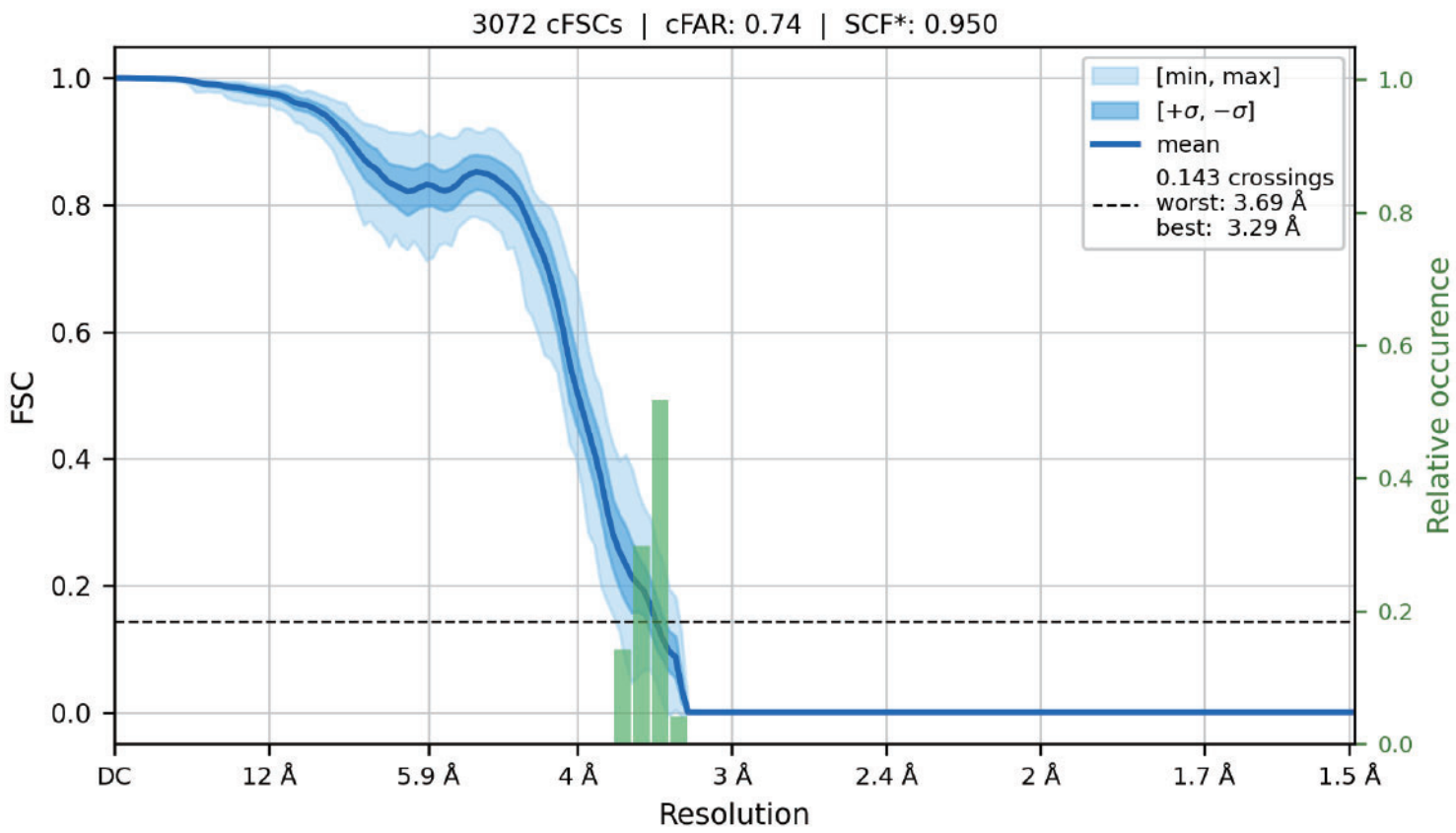

B **dDs:PaTP**

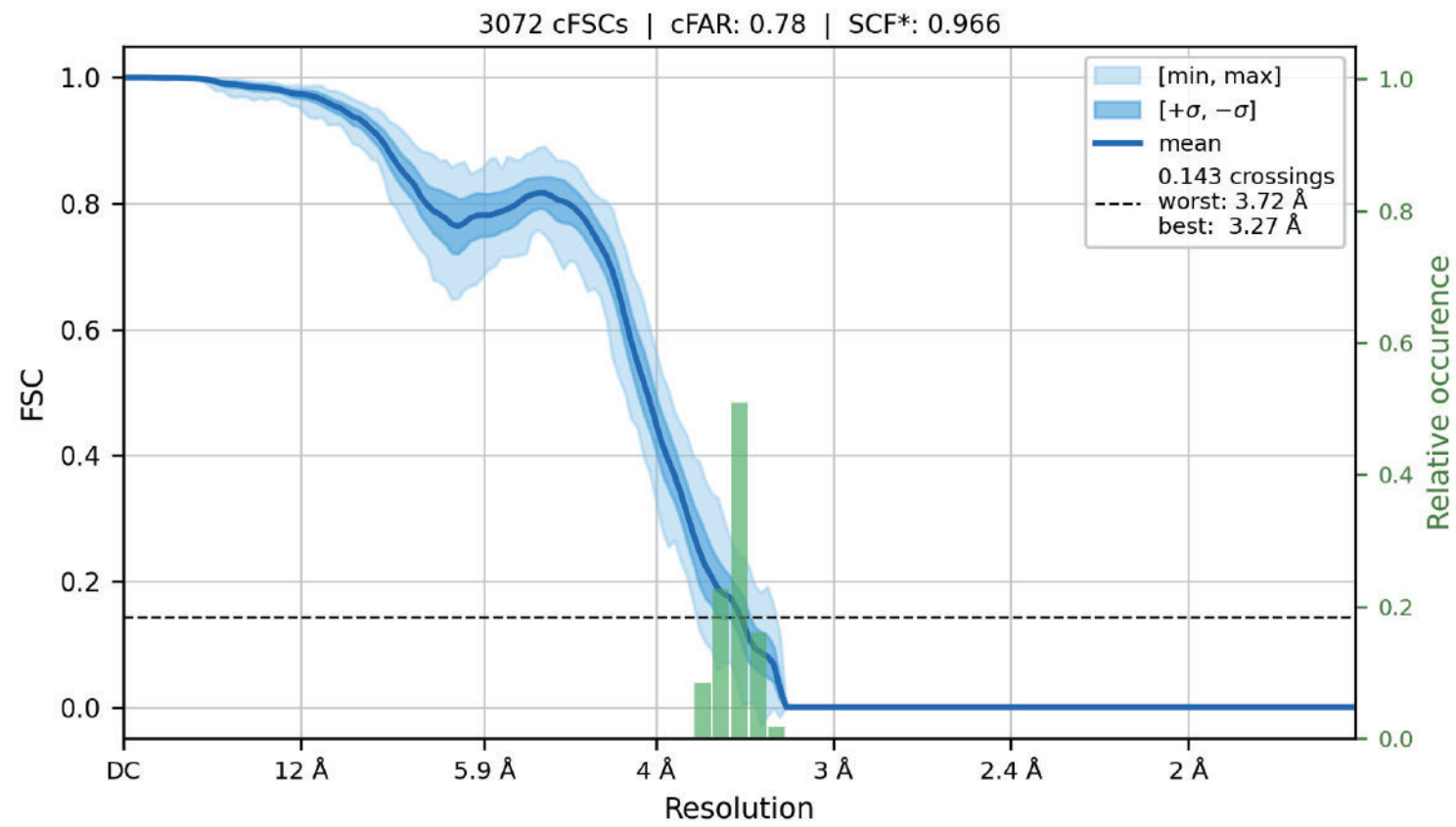

C **dPa:DsTP**

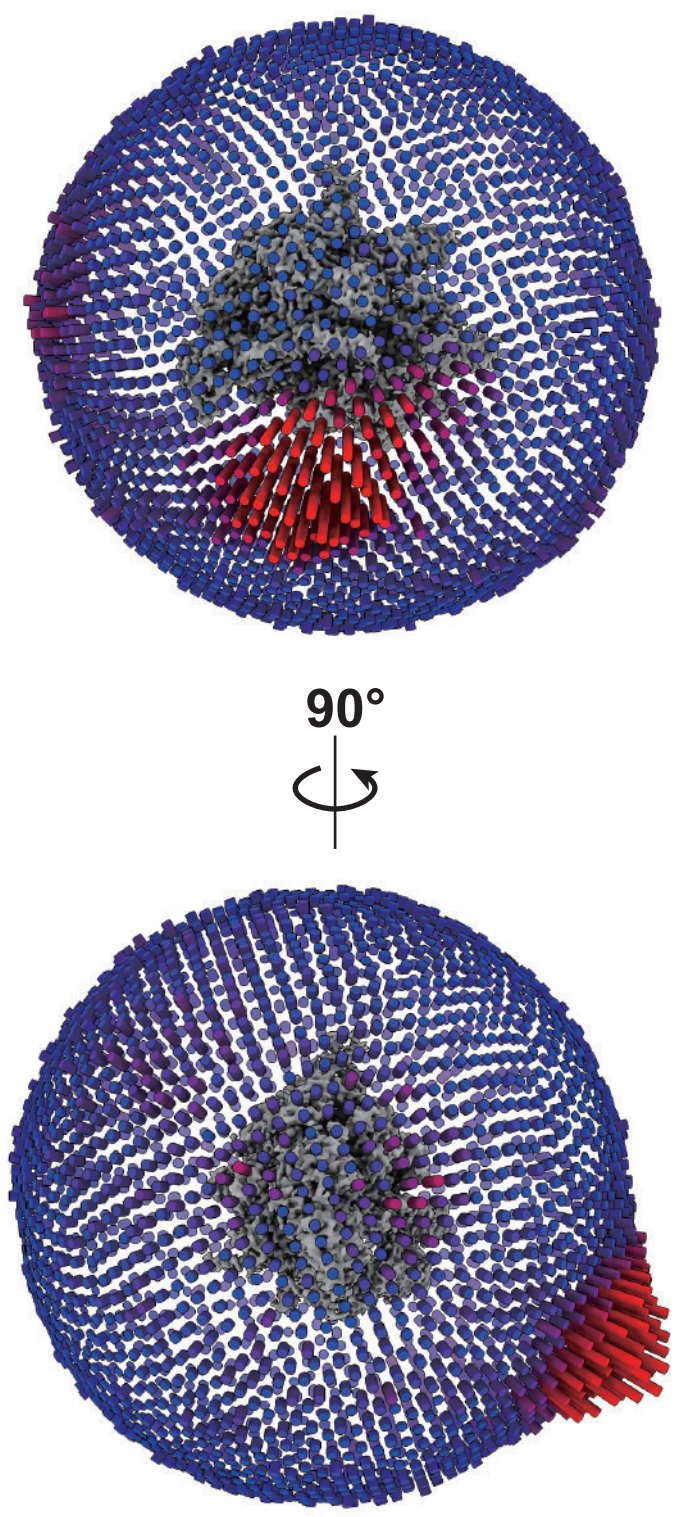

D **dDs:PaTP**

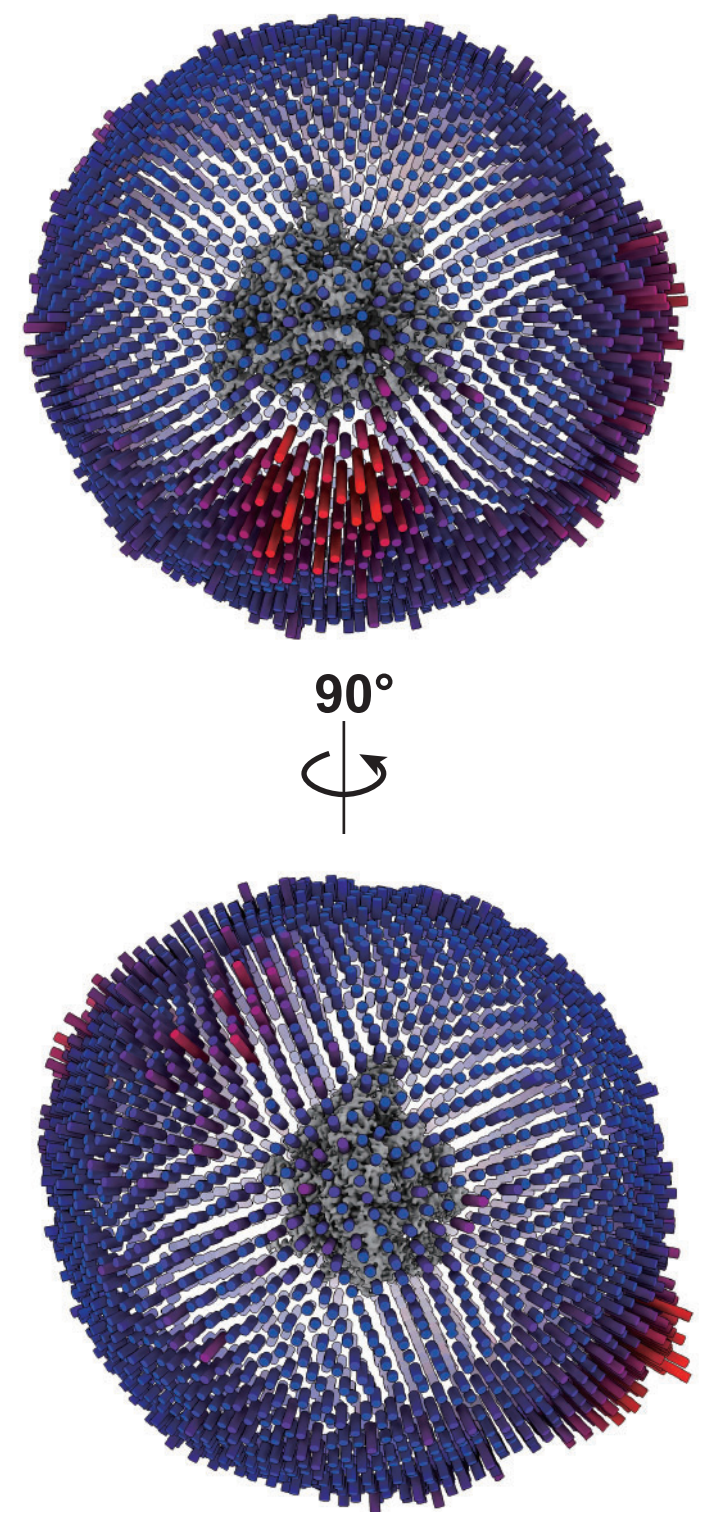

Table S1. Cryo-EM data collection, refinement and validation statistics

Dataset	dPa:DsTP	dDs:PaTP
**PDB**	10AG	10AN
**EMDB**	EMD-75025	EMD-75030
Microscope	Titan Krios G3i 300keV	Titan Krios G3i 300keV
Detector	Falcon 4i	K3
Cs (mm)	2.7	2.7
Pixel size (Å)	0.743	0.86
Electron dose (e-/Å^2)	30	30
Defocus range (µm)	-1.0 to -2.5	-1.0 to -2.5
Micrograph Number	12,618	7,620
**Reconstruction**		
Software	CryoSPARC v4.5	CryoSPARC v4.5
Particles refinement	23,770	30,356
Symmetry	C1	C1
Resolution (Å)	3.20	3.24
Sharpening B-factor (Å^2)	74.0	64.1
**Refinement**		
**Model composition**		
Number of atoms	25,930	25,929
Protein residues	3214	3214
Nucleotides	37	37
Ligand	6	5
**Bonds RMSD**		
Bonds lengths (Å)	0.0023	0.0046
Bonds angles (°)	0.48	0.54
**Validation**		
MolProbity score	1.35	1.53
Clash score	4.88	4.08
Rotamer outliers	0.84%	1.28%
C-beta outliers	0	0
**Ramachandran plot**		
Favored	97.56%	96.22%
Allowed	2.41%	3.75%
Outlier	0.03%	0.03%
